# SpoIIIL is a forespore factor required for efficient cell-cell signalling during *Bacillus subtilis* sporulation

**DOI:** 10.1371/journal.pgen.1011768

**Published:** 2025-07-03

**Authors:** Danae Morales Angeles, Kaitlyn Coleman, Chimezie Progress Odika, Chris L. B. Graham, Helena Chan, Michael Gilmore, Najwa Taib, Elda Bauda, Christine Moriscot, Benoit Gallet, Hannah Fisher, Per A. Bullough, Cécile Morlot, Darius Köster, Simonetta Gribaldo, Felipe Cava, Christopher D. A. Rodrigues

**Affiliations:** 1 School of Life Sciences, University of Warwick, Coventry, United Kingdom; 2 Australian Institute for Microbiology & Infection, University of Technology Sydney, Sydney Australia; 3 Department of Molecular Biology, Umea Universitet, Umea, Sweden; 4 Hub Bioinformatics and Biostatistics, Department of Computational Biology, Institut Pasteur, Paris, France; 5 Department of Molecular & Cellular Biology, Universite de Geneve, Geneva, Switzerland; 6 Institut de Biologie Structurale (IBS), University Grenoble Alpes, Grenoble, France; 7 School of Biosciences, The University of Sheffield, Sheffield, United Kingdom; 8 Centre for Mechanochemical Cell Biology, University of Warwick, Coventry, United Kingdom; 9 Department of Microbiology, Institut Pasteur, Paris, France; Tufts University School of Medicine, UNITED STATES OF AMERICA

## Abstract

During endospore formation, the mother cell and developing spore establish cell-cell signalling pathways that lead to compartment-specific transcription and key steps in morphogenesis. Endospore-forming bacteria also assemble a highly conserved essential membrane complex, called the A-Q complex, that physically connects these cells and may serve as a molecular conduit between them. While SpoIIIL was previously identified as a putative A-Q complex component in *Bacillus subtilis*, its exact role remains unclear. Here, we found that SpoIIIL does not function in the A-Q complex but instead acts as a forespore-specific factor required for efficient cell-cell signalling that leads to late mother cell transcription. Quantitative image analysis revealed that *spoIIIL* mutant spores do not exhibit hallmark phenotypes of A-Q complex mutants. Furthermore, unlike well-characterized A-Q complex proteins, SpoIIIL-GFP localizes uniformly in the forespore membrane before dispersing into the forespore cytoplasm. A synthetic sporulation screen identified a genetic relationship between *spoIIIL* and *murAB*, a paralog of *murAA*, required for efficient peptidoglycan precursor synthesis during sporulation. Cytological analysis indicates that the *spoIIIL murAB* double mutant is severely defective in the assembly of spore cortex peptidoglycan. Investigations into how SpoIIIL affects the cortex suggest it contributes to the activity of SpoIVB, a secreted forespore protease that initiates the signalling pathway required for processing of inactive pro-σK to active σK in the mother cell, which in turn up-regulates peptidoglycan precursor synthesis required for cortex formation. Accordingly, the *spoIIIL* mutant exhibits delayed and reduced pro-σK processing and decreased accumulation of peptidoglycan precursors. Thus, cortex assembly defects in the *spoIIIL murAB* double mutant results from alterations in separate pathways contributing to peptidoglycan precursor synthesis. Finally, phylogenetic analyses reveal that SpoIIIL is restricted to a subset of *Bacillales* species, highlighting evolutionary specialization in the signalling pathway leading to σK activation. Collectively, our findings redefine SpoIIIL as a forespore factor required for efficient cell-cell signalling that controls late mother-cell transcription.

## Author summary

Bacterial endospores allow bacteria to survive stress, persist in the environment and seed new and recurring infections. Cell-cell signalling is a cornerstone of how bacteria develop into spores, and the mother cell and developing spore establish several essential signalling pathways that underlie activation of compartment-specific σ factors at different stages of the development. In addition, communication between the mother cell and forespore involves the highly conserved A-Q complex, a specialized secretion-like system that, rather than directly regulating σ factor activation, plays a crucial role in sustaining forespore transcriptional potential. SpoIIIL was previously identified as a putative component of the A-Q complex, however its precise role remained unclear. Here we demonstrate that SpoIIIL does not function in the A-Q complex but instead contributes to the cell-cell signalling pathway that activates σK in the mother cell. Our findings refine the composition of the A-Q complex and provide new insight into the regulatory mechanisms controlling late mother-cell transcription.

## Introduction

Cell-cell signalling pathways allow organisms to coordinate transcriptional and morphological changes during development and differentiation. One well studied example of development is the process of bacterial endospore formation, known as sporulation, that occurs in many bacteria belonging to the *Firmicutes* phylum and includes the model organism *Bacillus subtilis* [1]. *B. subtilis* sporulation is induced by starvation and one of the earliest events in this process is the formation of a polar septum that results in two cells of unequal size, a larger mother cell and a smaller forespore [2]. Shortly after polar division, the mother cell membrane migrates around the forespore in a process called engulfment [2,3]. Upon engulfment membrane fission the forespore is surrounded by two membranes, an outer membrane derived from the mother cell and an inner membrane derived from the spore, and becomes isolated from the external environment [4]. Inside the mother cell the spore matures and develops a complex multilayered cell envelope, composed of specialized peptidoglycan (PG) called the cortex sandwiched between the two membranes, and a thick proteinaceous coat deposited on the exterior of the spore outer membrane [2,5]. Once the spore is mature, the mother cell lyses, releasing the spore into the environment where it remains dormant until nutrients become available [2].

The morphogenetic changes that occur during sporulation in *B. subtilis* are governed by the sequential activation of four σ factors, at different stages of development [2]. σF is activated in the spore shortly after polar division and leads to the production of proteins that signal σE activation in the mother cell [2]. σE-dependent transcription then leads to the production of proteins that facilitate key developmental events such as engulfment, coat assembly and σG activation in the spore [6,7]. Finally, σG activity in the forespore then signals σK activation in the mother cell [8]. σK activation plays an important role in the formation of the cortex, as it leads to transcriptional up-regulation of genes involved in PG precursors synthesis required for cortex assembly [9]. Importantly, σE and σK activation in the mother cell require signalling pathways that lead to cleavage of the inactive pro-σ forms, pro-σE and pro-σK, to their active forms. In both cases, signalling is initiated by proteins produced in the spore, under σF and σG, respectively [8,10,11]. In the case of σE, signalling from the forespore requires two proteins (SpoIIR and SpoIIT) to activate a membrane-bound protease (SpoIIGA) that cleaves pro-σE to σE [10–12]. In the case of σK, signalling is achieved through Regulated Intramembrane Proteolysis (RIP), where a site-1 protease produced in the spore (SpoIVB) leads to the activation of a membrane-embedded site-2 protease in the mother cell (SpoIVFB) that cleaves pro-σK to σK [13].

Interestingly, cell-cell communication between the mother cell and forespore also occurs through a physical connection established by a highly conserved specialized secretion-like complex, called the A-Q complex (also known as the feeding-tube) [14–17]. In *B. subtilis*, the A-Q complex is composed of nine σE-dependent proteins, eight encoded in the *spoIIIA* operon (*spoIIIAA-spoIIIAH*) and *gerM* gene, and a single forespore protein encoded by *spoIIQ* [14,18]. Several of the A-Q complex proteins exhibit structural similarity to proteins found in Type II, Type III and Type IV secretion systems, lending to the hypothesis that it may function as a hybrid specialized secretion system connecting the mother cell and forespore [14–16,19]. The exact identity of what is transported by the A-Q complex remains mysterious and several models have been put forth [14]. Although the exact function of this complex remains unclear, the absence of any A-Q complex proteins results in limited transcriptional potential in the forespore, including that associated with σG [15]. Furthermore, A-Q complex mutants also display morphological defects, and this includes rounder spores with deformed membranes [15].

The identification of SpoIIIL (previously known as YqzE) as an additional A-Q complex protein in the forespore raised further questions about the structure and function of this enigmatic complex [10]. The *spoIIIL* gene resides in the *comG* operon which encodes a Type IV pilus required for DNA uptake during competence [10,20]. Interestingly, *spoIIIL* transcription is regulated by two promoters: one by ComK, which upregulates *comG* transcription during competence and is situated upstream of *comGA*, and another σF-dependent promoter immediately upstream of *spoIIIL* [10,21,22]. Since Type IV pili are structurally similar to Type II secretion systems and given that the A-Q complex appears to be a hybrid specialized secretion-like complex, the identification of SpoIIIL led to the hypothesis that SpoIIIL may play a similar role in different secretion systems. Consistent with the idea that SpoIIIL could function in the A-Q complex, a *spoIIIL* mutant resulted in rounder forespores with reduced σG activity in a subpopulation of cells and this phenotype was exacerbated in a *spoIIIAH* mutant background [10].

In this study, we sought to define the role of SpoIIIL and establish whether it functions in the A-Q complex or in another pathway required for sporulation. Using a variety of cytological analysis, genetic screens and biochemical methods we found that SpoIIIL does not function in the A-Q complex. Instead, our data suggest that SpoIIIL is required for efficient cell-cell signalling at intermediate stages of development. We propose a model whereby SpoIIIL contributes to the activity of SpoIVB, the site-1 protease that initiates the RIP signalling pathway required for proteolytic activation of σK in the mother cell. Interestingly, while our data suggest that SpoIIIL plays a role in competence in *B. subtilis*, it does not always co-occur with the *comG* operon, suggesting divergent functions in other spore-forming Firmicutes. Collectively, our data redefine the role of SpoIIIL in sporulation and open the door for further studies examining how SpoIIIL could function as a moonlighting protein during distinct developmental processes.

## Results

### SpoIIIL is required for the oblong shape of the forespore and the timing of σG activity

**Fig 1:**
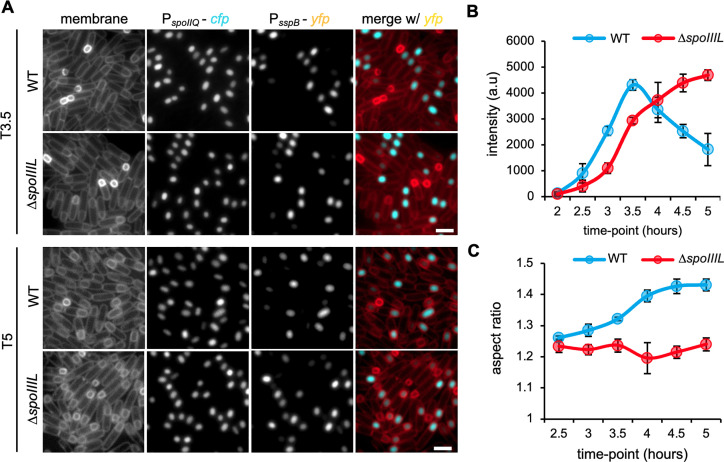
Forespore shape and timing of σG activity in WT and ∆*spoIIIL* mutant. (A) Representative images of WT and ∆*spoIIIL* mutant expressing CFP (under the control of a σF-dependent promoter) and YFP (under the control of a σG-dependent promoter) at T3.5 and T5. Merged images show membrane in red and YFP in cyan. Scale bar is 2 μm. (B) Mean intensity (errors bars are ± STDEV of 3 biological replicates; n > 500, for each biological replicate) of σG-dependent YFP signal in WT and ∆*spoIIIL* mutant forespores, during a sporulation time-course. (C) Mean forespore aspect ratio (length/width) (errors bars are ± STDEV of 3 biological replicates; n > 500, for each biological replicate) of WT and ∆*spoIIIL* mutant forespores, during a sporulation time-course.

Previous work proposed SpoIIIL as a component of the A-Q complex [10]. This role for SpoIIIL was based on microscopy data showing that the Δ*spoIIIL* mutant exhibits hallmark phenotypes of A-Q complex mutants (reduced σG activity and rounder spores) [10]. To better analyze the *spoIIIL* phenotypes, we developed a strain to quantitatively monitor spore shape and σG activity in the same cell (Fig 1A). This strain harbored two fluorescent reporters in the forespore compartment: a CFP reporter produced under the control of σF that allows examination of spore shape from early during developmental stages (P_*spoIIQ*_-*cfp*) and a YFP reporter produced under the control of σG that allows measurement of σG activity (P_*sspB*_-*yfp)*. Using these reporters, we examined forespore shape and σG activity in WT and the Δ*spoIIIL* mutant over a sporulation time-course (Fig 1A). Consistent with previous work [23], WT forespores became more oblong overtime (Fig 1C). Furthermore, and consistent with previous results [15,18,24], σG activity in WT forespores peaked after engulfment completion at T3.5 (Fig 1B). As expected, the Δ*spoIIIL* mutant forespores were rounder than WT (Figs 1C and S1A). On the other hand, the Δ*spoIIIL* mutant forespores exhibited a slight delay in σG activity, but reached WT σG activity levels at T4.5 (Fig 1B), suggesting that SpoIIIL’s role is not related to the A-Q complex.

### SpoIIIL localizes uniformly in the forespore membrane and undergoes a change in localization during spore maturation

**Fig 2:**
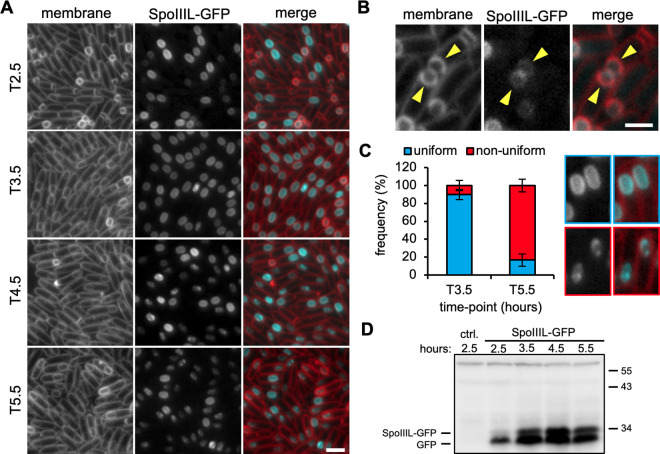
SpoIIIL localization during early-to-intermediate stages of sporulation. (A) Representative images of SpoIIIL-GFP during a sporulation time-course. Membrane in merged image is shown in red and SpoIIIL-GFP in cyan. Scale bar is 2 μm. (B) Representative image showing that SpoIIIL-GFP does not become enriched in the engulfing membrane (pointed out by yellow arrowheads). Scale bar is 2 μm. (C) Bar graph (left) showing the mean proportion of uniform (in blue) and non-uniform (in red) SpoIIIL-GFP localization at T3.5 and T5.5 (error bars are ± STDEV of 3 biological replicates; n > 100, for each biological replicate). Representative examples (right) of sporangia with uniform (outlined in blue) and non-uniform (outlined in red) localization of SpoIIIL-GFP. (D) Immunoblot analysis of cell lysates containing SpoIIIL-GFP, collected during a sporulation time-course. SpoIIIL-GFP was immuno-detected using anti-GFP antibodies. The positions of SpoIIIL-GFP and GFP are indicated to the left and position of molecular weight markers to the right.

Our data above suggest that SpoIIIL’s function is unrelated to the A-Q complex. If this is the case, then SpoIIIL may exhibit a different localization than SpoIIQ, a well-defined A-Q complex protein produced in the forespore that becomes localized in the septal membrane facing the mother cell during engulfment [18,25,26]. We generated a functional SpoIIIL-GFP fluorescent-fusion (S1B and S1C Fig) and examined its localization during a sporulation time-course (Fig 2A). At early stages of development (T2.5), SpoIIIL-GFP localized uniformly in the forespore inner membrane, with no specific enrichment in the forespore engulfing septal membrane facing the mother cell (Fig 2A and 2B). The uniform localization of SpoIIIL-GFP in the forespore inner membrane was maintained after engulfment completion (Fig 2A) and was comparable to the localization of a GFP-fusion to two transmembrane segments of MalF (integral membrane protein involved in maltose import in *Escherichia coli*; MalF-GFP) (S1F Fig). Furthermore, although protein topology prediction tools suggest that SpoIIIL is a cytoplasmic protein (Phobius) [27], SpoIIIL contains a series of hydrophobic residues towards the N-terminus and C-terminus, (S1D Fig) which may contribute to its membrane localization. Consistent with this idea, computational simulations of SpoIIIL on a 2D planar membrane suggest that SpoIIIL remains membrane associated over a nanoscale time frame (S1D Fig).

Interestingly, as forespores progressed through development, SpoIIIL-GFP changed localisation. The SpoIIIL-GFP signal became less uniform and could be observed as blotchy patches near the membrane or in the forespore cytoplasm (Fig 2A). For simplicity, we designated this less uniform signal as non-uniform. A similar change in localization was observed for an almost fully functional fluorescent fusion with GFP at the N-terminus of SpoIIIL (GFP-SpoIIIL) (S1B and S1E Fig). Quantification of the number of forespores with uniform and non-uniform signal at two time-points, T3.5 and T5.5, confirmed the change in SpoIIIL-GFP localization: at T3.5, 89.8% of spores displayed uniform SpoIIIL-GFP signal, while at T5.5 the fraction of spores with uniform signal decreased to 16.8% (Fig 2C). Immunoblot analysis suggests that the change in SpoIIIL-GFP localization between T3.5 and T5.5 is not due to changes in protein levels, as SpoIIIL-GFP levels were maintained across these time-points (Fig 2D). These results indicate that SpoIIIL undergoes a change in localization during spore maturation.

### A synthetic sporulation screen identifies a relationship between *spoIIIL* and genes involved in the assembly of the spore envelope

**Fig 3:**
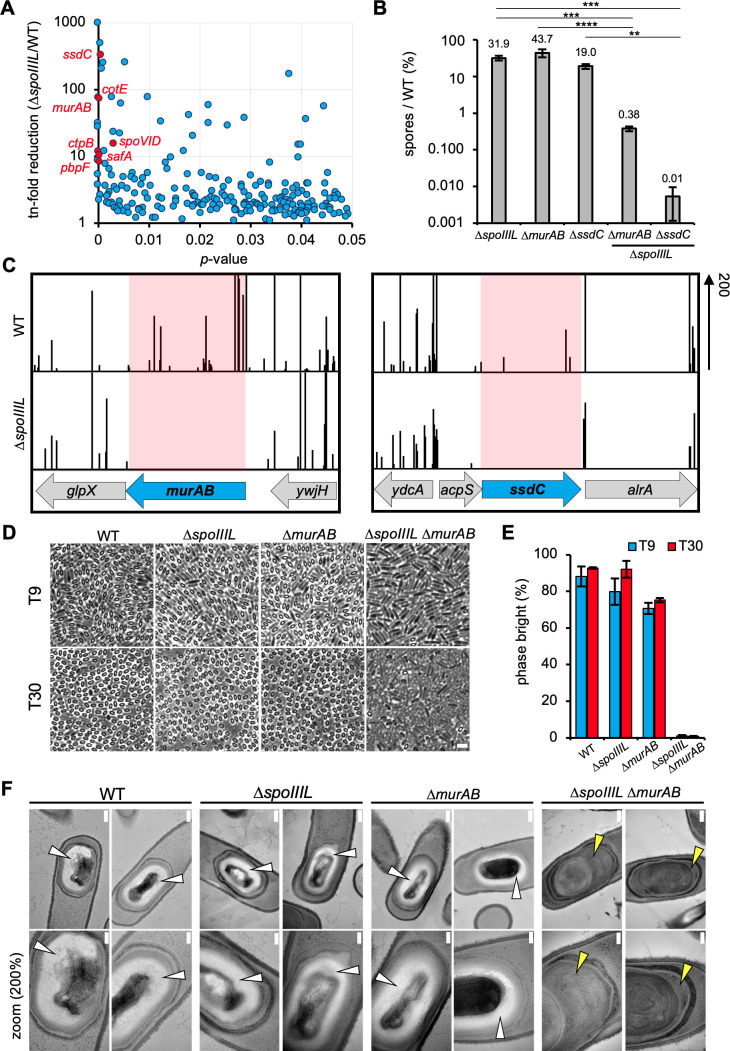
Genetic relationship between *spoIIIL* and spore envelope genes revealed by Tn-seq and cytological analysis. (A) Scatterplot showing fold-reduction of transposon insertions in ∆*spoIIIL* relative to WT, with corresponding *P* values. Genes involved in PG synthesis (*murAB*, *pbpF*), spore coat (*safA*, *spoVID* and *cotE*), cortex (*ssdC*) and σ^K^ activation (*ctpB*) with high fold-reduction and low *P* value are labelled and colored red. (B) Sporulation efficiency of ∆*spoIIIL,* ∆*murAB* and ∆*ssdC* single mutants and ∆*spoIIIL* ∆*murAB* and ∆*spoIIIL* ∆*ssdC* double mutants (n = 3, mean ± STDEV; **p < 0.01, ***p < 0.001, ****p < 0.0001, Tukey’s multiple comparisons test). (C) Tn-seq profiles at the *murAB* and *ssdC* locus of WT and ∆*spoIIIL* cells following 30 h of growth and sporulation in exhaustion medium. The height of the vertical lines represents the number of Tn-seq reads at each position (scale is 200 sequencing reads). Shaded regions highlight the significant reduction in sequencing reads at the *murAB* and *ssdC* locus. (D) Representative phase contrast images showing spore refractivity at 9 (T9) and 30 (T30) hours after the onset of sporulation in WT and in ∆*spoIIIL,* ∆*murAB* and ∆*spoIIIL* ∆*murAB* mutant sporangia. Scale bar is 2 μm. (E) Mean frequency of phase bright spores in the WT and ∆*spoIIIL,* ∆*murAB* and ∆*spoIIIL* ∆*murAB* mutants (error bars are ± STDEV of 3 biological replicates; n > 100, for each biological replicate). (F) Representative electron micrographs of sporangia sections in the WT and ∆*spoIIIL,* ∆*murAB* and ∆*spoIIIL* ∆*murAB* mutants. Scale bar is 200 nm and 100 nm in standard and zoomed-in images, respectively. White arrowheads point to a distinct light grey cortex. Yellow arrowheads point to amorphous grey material, reflecting a cortex synthesis defect.

The data above suggest that SpoIIIL does not function in the A-Q complex. To identify genes that function with SpoIIIL during sporulation, we used transposon-sequencing (Tn-seq) combined with sporulation heat-kills to screen for genes that become critical for sporulation in its absence. Saturated transposon libraries were constructed in the WT and the Δ*spoIIIL* mutant (see Materials & Methods). Analysis of the transposon insertion profiles revealed a set of genes in which insertions were underrepresented in the Δ*spoIIIL* mutant when compared to the WT (Fig 3A and 3C) (S1 Table). Interestingly, many of the genes identified encode proteins with roles in the assembly of various spore envelope layers, suggesting a genetic relationship between SpoIIIL and the spore envelope (S1 Table). These include genes involved in the spore coat (*safA, spoVID* and *cotE*), germ cell wall (*pbpF*), spore shape and cortex (*ssdC*, Fig 3C) and PG precursor synthesis (*murAB*, Fig 3C) [5,23,28–30]. Deleting most of these genes in combination with a Δ*spoIIIL* mutation only caused a moderate decrease in sporulation efficiency relative to the Δ*spoIIIL* mutant (S1 Table). By contrast, combining Δ*ssdC* or Δ*murAB* with Δ*spoIIIL* resulted in pronounced sporulation defects, with sporulation efficiencies of 0.01% and 0.38%, respectively (Fig 3B). Prompted by the severity of the Δ*spoIIIL* Δ*murAB* and Δ*spoIIIL* Δ*ssdC* double mutant sporulation defect, we investigated these mutants further.

### Sporangia lacking SpoIIIL and MurAB exhibit a severe cortex synthesis defect

Our previous work indicated that SsdC is required for forespore shape and for proper cortex development, with the Δ*ssdC* mutant resulting in phase bright spores with denser and less uniform cortex [23]. MurAB on the other hand has been shown to be required for efficient PG precursor synthesis which promotes efficient engulfment and may also promote efficient cortex synthesis [28]. Based on these observations, we reasoned that the sporulation defects of the Δ*spoIIIL* Δ*ssdC* and Δ*spoIIIL* Δ*murAB* double mutants may result from more pronounced defects in forespore shape, engulfment and/or cortex assembly.

To test this, we first examined whether the Δ*spoIIIL* Δ*ssdC* and Δ*spoIIIL* Δ*murAB* double mutants exhibit a more pronounced defect in spore shape, engulfment and σG activity compared to Δ*spoIIIL*, by examining the mutants in the CFP/YFP reporter background during a time-course covering early-to-intermediate stages of sporulation (S2 Fig). The Δ*spoIIIL* Δ*ssdC* double mutant produced rounder forespores compared to either of the single mutants suggesting that the absence of SsdC exacerbates the shape defect of Δ*spoIIIL* (Figs S2D and 2E). Furthermore, σG activity reached similar levels in the Δ*spoIIIL* Δ*ssdC* double mutant as the Δ*ssdC* mutant, although following a similar delay as observed for the Δ*spoIIIL* mutant (S2C and S2E Fig). Engulfment completion (S2H Fig) and spore shape (S2G Fig) was comparable between Δ*spoIIIL* Δ*murAB* and Δ*murAB*. Interestingly, the Δ*murAB* mutant also exhibited a slight delay in σG activation and did not reach WT levels (S2F Fig). The Δ*spoIIIL* Δ*murAB* double mutant on the other hand, although more delayed than either of the single mutants, eventually reached WT levels of σG activity (S2F Fig). Thus, while the Δ*spoIIIL* Δ*ssdC* and Δ*spoIIIL* Δ*murAB* double mutants exhibit mild differences in spore shape and σG activity relative to their single mutant counterparts, these differences are unlikely to be the major cause of their pronounced sporulation defect.

Next, we examined whether the Δ*spoIIIL* Δ*ssdC* and Δ*spoIIIL* Δ*murAB* exhibit a more pronounced defect in cortex synthesis, which results in a change in forespore refractivity, from phase dark to phase bright, as they mature [23]. Interestingly, while the Δ*spoIIIL* Δ*ssdC* double mutant produced phase-bright spores (S2A Fig), the Δ*spoIIIL* Δ*murAB* double mutant produced virtually none (Fig 3D). Quantification showed that the WT and Δ*spoIIIL* mutant produced similar amounts of phase bright spores (T30; 88% and 80%, respectively) (Fig 3E). The Δ*murAB* mutant on the other hand produced fewer phase-bright spores compared to WT (70.6%) (Fig 3E), suggesting that PG precursor synthesis by MurAB also contributes to efficient cortex synthesis. Recapitulating the microscopy data, the Δ*spoIIIL* Δ*murAB* double mutant only produced 0.8% phase-bright spores, corresponding to a 100-fold reduction in phase bright spores compared to WT, similar to its sporulation efficiency (Figs 3E and S2B). Transmission electron microscopy (TEM) confirmed the absence of a distinct light grey cortex in the Δ*spoIIIL* Δ*murAB* double mutant and instead an amorphous grey material was observed (Fig 3F). Together, these data suggest that SpoIIIL is required for cortex synthesis in cells lacking MurAB.

### SpoIIIL is required for efficient accumulation of PG precursors at intermediate stages of development

**Fig 4:**
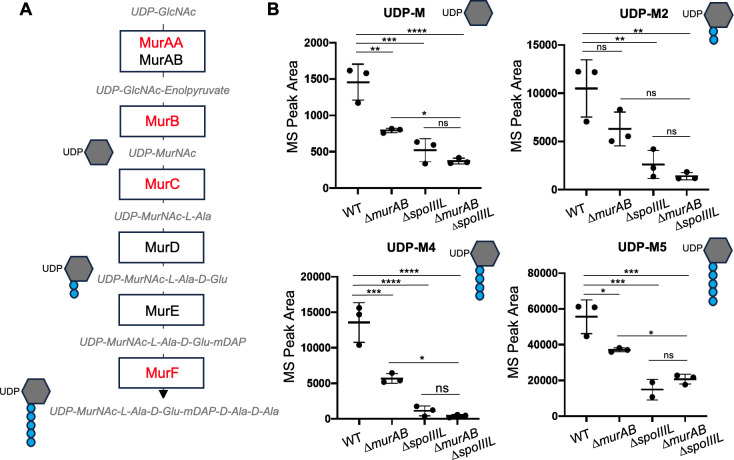
PG precursor levels in the absence of SpoIIIL and MurAB. (A) Steps in PG precursor biosynthesis leading up to the production of UDP-MurNAc-L-Ala-D-Glu-mDAP-D-Ala-D-Ala (UDP-M5) by MurF. The proteins labelled in red have been shown to be upregulated by σK (see text). (B) Peptidoglycan precursor levels in WT, ∆*murAB,* ∆*spoIIIL* and ∆*murAB* ∆*spoIIIL* at 4.5 hours after the onset of sporulation (T4.5). Four PG precursors were identified by LC-MS: UDP-MurNAc (UDP-M), UDP-MurNAc-L-Ala-D-Glu (UDP-M2), UDP-MurNAc-L-Ala-D-Glu-mDAP-D-Ala (UDP-M4) and UDP-MurNAc-L-Ala-D-Glu-mDAP-D-Ala-D-Ala (UDP-M5). UDP-M4 is not synthesized but is likely a by-product of the cleavage of UDP-M5 by an unknown enzyme. (n = 3, mean ± STDEV; *p < 0.05, **p < 0.01, ***p < 0.001, ****p < 0.0001, Tukey’s multiple comparisons test).

The data above indicate that sporulating cells lacking SpoIIIL are sensitive to mild defects in PG synthesis, with the Δ*spoIIIL* Δ*murAB* double mutant producing very few phase-bright spores with a mature cortex. How could the combined absence of SpoIIIL and MurAB impact cortex synthesis? Although the cortex PG synthases SpoVD and SpoVE are localized in the forespore outer membrane throughout engulfment, cortex synthesis only occurs post-engulfment with σK activation in the mother-cell and subsequent transcriptional upregulation of a subset of genes required for production of PG precursors (*murAA*, *murB*, *murC* and *murF*; Fig 4A) [9]. Since MurAB plays a direct role in maintaining efficient PG precursor levels during sporulation [28], but most sporangia lacking MurAB still assemble a cortex (Fig 3E), we hypothesized that SpoIIIL indirectly influences the upregulation of PG precursors such that the absence of both proteins would affect PG precursor levels and thereby compromise cortex assembly. To test this, we examined PG precursor levels in WT and Δ*spoIIIL* mutant cells by liquid chromatography-mass spectrometry (LC-MS) analysis. We were able to confidently quantify four PG precursors: UDP-MurNAc, UDP-MurNAc-L-Ala-D-Glu (UDP-M2), UDP-MurNAc-L-Ala-D-Glu-mDAP-D-Ala (UDP-M4) and MurNAc-L-Ala-D-Glu-mDAP-D-Ala-D-Ala (UDP-M5) (Fig 4B). In all cases, PG precursor levels were significantly reduced in the Δ*spoIIIL* mutant compared to WT (Fig 4B). Furthermore, although variation between biological replicates compromised statistical significance, there was a reduction in the mean values for three of the four PG precursors examined in the Δ*spoIIIL* Δ*murAB* double mutant, relative to either of the single mutants (Fig 4B). These data suggest that SpoIIIL is required for efficient accumulation of PG precursors and that the Δ*spoIIIL* Δ*murAB* cortex phenotype results from defects in separate pathways that contribute to PG precursor synthesis.

### SpoIIIL is required for efficient SpoIVB activity and pro-σK processing

**Fig 5:**
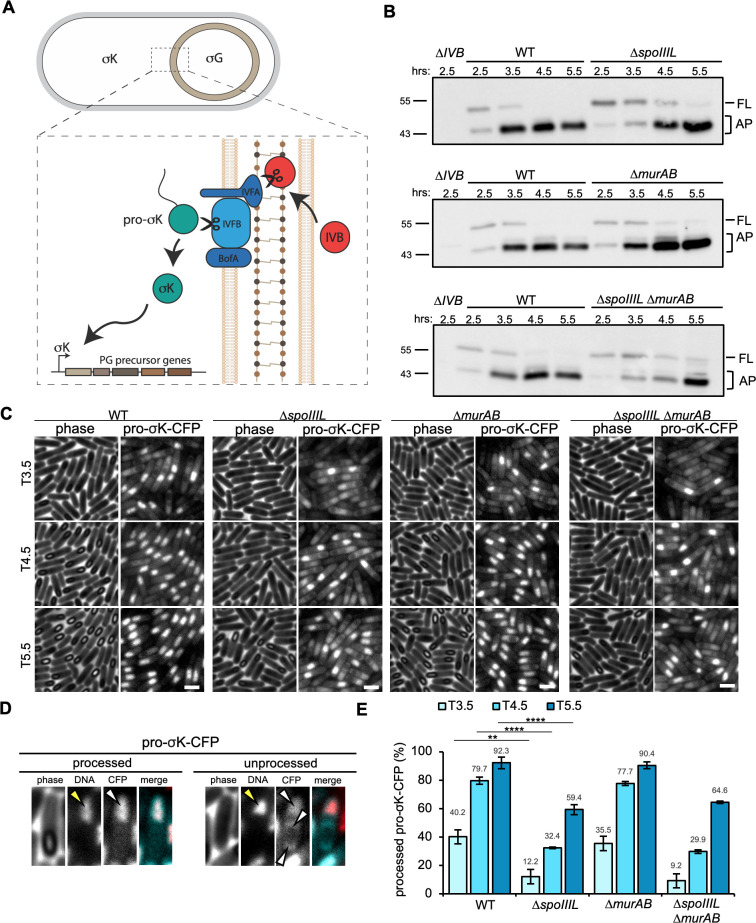
SpoIVB activity and σK activation in the absence of SpoIIIL. (A) Activation of σK is controlled by a signalling pathway across the forespore membranes that involves Regulated Intramembrane Proteolysis (RIP) of an inactive form of σK (pro-σK) to its active form (σK). This RIP signalling pathways involves the forespore produced Site-1 protease, SpoIVB, and the mother cell produced Site-2 protease, SpoIVFB, which cleaves pro-σK to σK. SpoIVFB activity is inhibited by SpoIVFA (and BofA) until the SpoIVB protease is produced and secreted into the intermembrane space, where it then cleaves SpoIVFA, releasing the inhibition on SpoIVFB and promoting processing of pro-σK to σK. Binding of σK to the promoter of genes required for PG precursor synthesis, leads to increased PG precursor synthesis required for cortex PG synthesis. (B) Immunoblots comparing SpoIVB activity in WT and ∆*spoIIIL,* ∆*murAB and* ∆*spoIIIL* ∆*murAB* mutants. Full-length (FL) and autoproteolyzed (AP) forms of SpoIVB are pointed out on the right and the position of molecular weight markers are shown to the left. For uncropped blots showing lower molecular weight autoproteolytic forms of SpoIVB refer to S3B Fig. (C) Representative images of pro-σK-CFP localization at 3.5 (T3.5), 4.5 (T4.5) and 5.5 (T5.5) hours after the onset of sporulation in WT and ∆*spoIIIL,* ∆*murAB and* ∆*spoIIIL* ∆*murAB* mutant backgrounds. Scale bar is 2 μm. For the same image panels showing the DNA and merge refer to S3E Fig. (D) Representative example of processed (cleaved) and unprocessed (uncleaved) pro-σK-CFP, used as the basis of processed pro-σK-CFP quantification. Yellow arrowheads point to the mother cell nucleoid. White arrowheads point to the location of the pro-σK-CFP signal. (E) Mean frequency of processed pro-σK-CFP at 3.5, 4.5 and 5.5 hours after the onset of sporulation in WT and ∆*spoIIIL,* ∆*murAB and* ∆*spoIIIL* ∆*murAB* mutant backgrounds (errors bars are ± STDEV of 3 biological replicates, > 100 cells for each biological replicate; **p < 0.01, ****p < 0.0001, Tukey’s multiple comparisons test).

As SpoIIIL is produced in the forespore, one way in which it could contribute to maintaining sufficient PG precursor levels for cortex synthesis is by influencing the function of the forespore-produced, secreted protease, SpoIVB which initiates the RIP signalling pathway that leads to σK activation (Fig 5A) [13]. Thus, we first tested if SpoIIIL influences *spoIVB* transcription and SpoIVB levels.

To examine *spoIVB* transcription, we fused the *spoIVB* promoter to *cfp* (P_*spoIVB*_-*cfp*) and monitored CFP signal at 4.5 and 5.5 hours after the onset of sporulation (T4.5 & T5.5), which coincides with the window of SpoIVB activity [31]. Quantitative analysis of CFP signal suggests that *spoIVB* transcription is similar between the WT and Δ*spoIIIL* mutant, indicating that SpoIIIL does not influence *spoIVB* transcription (S3C and S3D Fig). To examine SpoIVB levels, we used immunoblotting. SpoIVB levels were examined at four time-points after the onset of sporulation (T2.5, T3.5, T4.5 and T5.5) that coincide with the activation of σG which is required for efficient transcription of SpoIVB, and SpoIVB levels, that signal pro-σK processing [8,32,33]. SpoIVB is an autoproteolytic serine peptidase and its activity leads to its autoproteolysis overtime, resulting in the formation of proteolyzed forms that by immunoblot appear at a lower molecular weight than the full-length protein [32–34]. Consistent with previous reports [32–34], in WT cells we predominantly observed 2 bands, a fainter band that corresponds to full-length SpoIVB (FL) and another darker band that corresponds to autoproteolyzed SpoIVB (AP) (Fig 5B). Importantly, in WT cells SpoIVB was almost completed proteolyzed by T4.5 (Fig 5B). In the Δ*spoIIIL* mutant however, we observed a band corresponding to full-length SpoIVB across all time points examined (Fig 5B), suggesting that SpoIVB autoproteolysis is incomplete and that SpoIIIL is required for efficient SpoIVB activity. Similar observations were made in Δ*spoIIIL* Δ*murAB* double mutant (Fig 5B). In the Δ*murAB,* although a faint band of full-length SpoIVB was observed at all time-points, there was a higher degree of SpoIVB autoproteolysis than in the Δ*spoIIIL* mutant and this was particularly evident at T3.5 and T4.5 (Fig 5B).

To test if the decreased SpoIVB activity observed in the Δ*spoIIIL and* Δ*spoIIIL* Δ*murAB* mutants affects σK activation, we examined and quantified the frequency of pro-σK processing during a sporulation time-course that coincides with the window of SpoIVB activity and σK activation, using a previously characterized pro-σK-CFP fluorescent fusion [35]. Pro-σK-CFP initially appears as a diffuse cytoplasmic signal in the mother cell (Fig 5D; unprocessed). As a result of SpoIVB-dependent signalling, pro-σK-CFP is cleaved by SpoIVFB and then co-localizes with the mother cell nucleoid forming a bright CFP patch that is consistent with its proteolytic processing and association with core RNA polymerase (Fig 5D; processed) (Fig 5C) [36]. Importantly, in the Δ*spoIVB* mutant pro-σK-CFP fails to produce bright CFP patches on the nucleoid and instead appears as a patchy cytoplasmic signal in the mother cell [36] (S3A Fig). In WT and Δ*murAB* cells, bright CFP patches could be observed in many cells at T3.5 and increased in similar frequency by T5.5 (Fig 5C and 5E). In the Δ*spoIIIL* and Δ*spoIIIL* Δ*murAB* double mutant however, we observed fewer cells with bright CFP patches across all time-points (Fig 5C and 5E). By T5.5, only 59.4% and 64.6% of the cells had bright CFP patches in Δ*spoIIIL* and Δ*spoIIIL* Δ*murAB,* respectively. For comparison, bright CFP patches were observed in 92.3% and 90.4% in the WT and Δ*murAB* mutant, respectively. These data suggest that pro-σK-CFP processing is delayed and less efficient in the absence of SpoIIIL and that Δ*spoIIIL* Δ*murAB* double mutant cortex phenotype results from defects in separate pathways that contribute to PG precursor synthesis: a σK-dependent pathway involving SpoIIIL and σK-independent pathway involving MurAB.

### Sporangia lacking SpoIIIL and CtpB exhibit a subtle delay in the timing of pro-σK processing

One gene in which transposon insertions were underrepresented in the Δ*spoIIIL* mutant was *ctpB* (S4B Fig) (S1 Table). CtpB is serine protease required for efficient cleavage of SpoIVFA, thereby fine-tuning efficient SpoIVFB activation and pro-σK processing (S4A Fig) [35,37,38]. Confirming the Tn-seq data, while the Δ*ctpB* mutant exhibited a mild sporulation defect (79.8%), the Δ*ctpB* Δ*spoIIIL* double mutant only produced 6.3% spores (S4D Fig). Since the above data suggest that SpoIIIL is required for efficient pro-σK processing, it seemed likely that Δ*spoIIIL* Δ*ctpB* would exhibit a more pronounced defect in pro-σK processing compared to either of the single mutants. To test this, we compared pro-σK processing using the pro-σK-CFP fluorescent fusion mentioned above, in the Δ*ctpB,* Δ*spoIIIL* and Δ*spoIIIL* Δ*ctpB* mutants (S4C Fig). Consistent with previous reports showing that CtpB contributes to the timing of pro-σK processing [35], in the Δ*ctpB* mutant we observed fewer cells with bright CFP patches at T3.5 compared to WT: 15.7% versus 36.2%, respectively (S4E Fig). At T4.5 and T5.5 pro-σK processing was similar between the WT and Δ*ctpB* mutant (S4E Fig). Similarly, compared to the Δ*spoIIIL* mutant, the Δ*spoIIIL* Δ*ctpB* double mutant had fewer cells with bright CFP patches at T3.5 and T4.5 (S4E Fig): 4.7% vs 1.8% and 24.3% vs 14.2%, in the Δ*spoIIIL* mutant vs Δ*spoIIIL* Δ*ctpB* double mutant, respectively. However, by T5.5 pro-σK processing was similar between the Δ*spoIIIL* mutant and Δ*spoIIIL* Δ*ctpB* double mutant (S4E Fig). Thus, the absence of CtpB results in a subtle enhancement of the Δ*spoIIIL* mutant pro-σK processing defect. However, since we observed the same number of cells with processed pro-σK-CFP at T5.5 in the Δ*spoIIIL* and Δ*spoIIIL* Δ*ctpB* mutants but that Δ*spoIIIL* Δ*ctpB* yields fewer heat-resistant spores than the Δ*spoIIIL* (S4D Fig), we conclude that CtpB likely plays an additional role in maintaining sporulation in the absence of SpoIIIL. Nonetheless, these data provide additional evidence that SpoIIIL functions in the cell-cell signalling pathway that leads to σK activation.

### The change in SpoIIIL localization at intermediate stages of spore development depends on cortex PG synthesis

**Fig 6:**
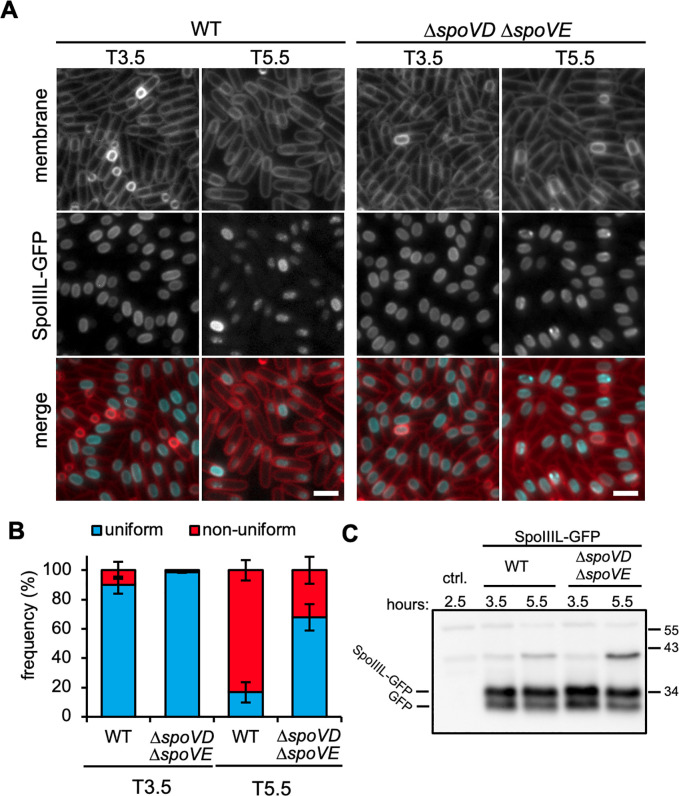
SpoIIIL-GFP localization in sporangia lacking the cortex synthases, SpoVD and SpoVE. (A) Representative images of SpoIIIL-GFP in otherwise WT and in the ∆*spoVD* ∆*spoVE* double mutant. Membrane in merged image is shown in red and SpoIIIL-GFP in cyan. Scale bar is 2 μm. (B) Bar graph showing the mean frequency of uniform (in blue) and non-uniform (in red) SpoIIIL-GFP localization at T3.5 and T5.5 in WT and in the ∆*spoVD* ∆*spoVE* double mutant (errors bars are ± STDEV of 3 biological replicates; n > 100, for each biological replicate). (C) Immunoblot analysis of cell lysates containing SpoIIIL-GFP in WT and in the ∆*spoVD* ∆*spoVE* double mutant, collected at T3.5 and T5.5. SpoIIIL-GFP was immuno-detected using anti-GFP antibodies. The positions of SpoIIIL-GFP and GFP are indicated to the left and position of molecular weight markers to the right.

Our data suggest that the absence of SpoIIIL leads to a decrease in PG precursor synthesis, thus affecting cortex synthesis. Since the change in SpoIIIL-GFP localization at intermediate stages of development coincides with the timing of cortex synthesis, we wanted to know if the change in SpoIIIL’s localisation depends on the cortex. To this end, we examined SpoIIIL-GFP localization in cells lacking the cortex synthases SpoVD and SpoVE at 3.5 and 5.5 hours after the onset of sporulation (T3.5 and T5.5) (Fig 6). These time-points were chosen because they show clear changes in SpoIIIL-GFP localization from uniform to non-uniform in WT cells. (Fig 2A and 2C). In the absence of SpoVD and SpoVE, SpoIIIL-GFP did not undergo a change in localization from uniform to non-uniform to the same extent as WT (Fig 6A). Like WT, most forespores lacking SpoVD and SpoVE exhibited uniform SpoIIIL-GFP at T3.5 (Fig 6A and 6B). At T5.5 however, and unlike the WT, most forespores in the Δ*spoVD* Δ*spoVE* double mutant displayed uniform SpoIIIL-GFP localization: 67.8% vs 16.8% in Δ*spoVD* Δ*spoVE* and WT, respectively (Fig 6B). Immunoblot analysis suggests that SpoIIIL-GFP levels are comparable between the WT and Δ*spoVD* Δ*spoVE* double mutant (Fig 6C). These data suggest that cortex synthesis by SpoVD and SpoVE is required for the change in SpoIIIL-GFP localization.

### *spoIIIL* is exclusively found within the *Bacillales* and its co-occurrence with the ComG operon is species-specific

**Fig 7:**
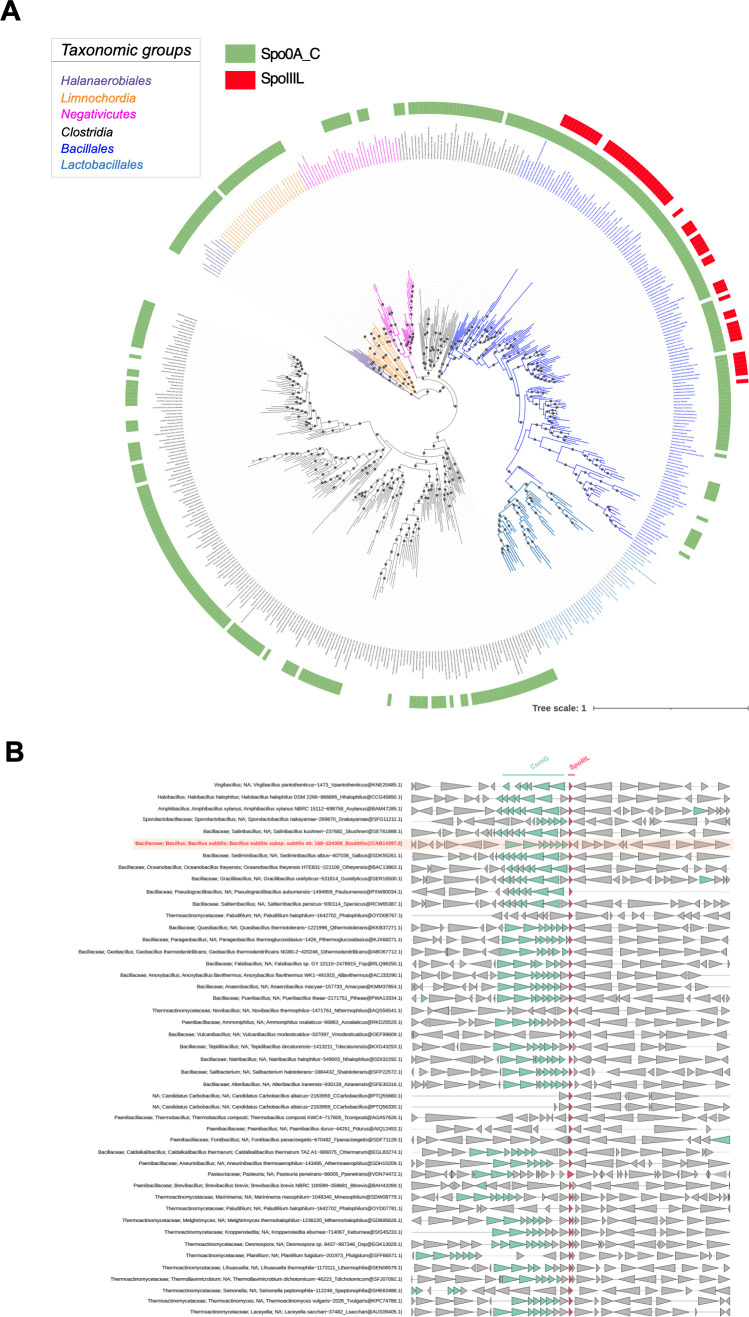
SpoIIIL phylogenetic distribution within the Firmicutes and co-occurrence with ComG operon. (A) Phylogenetic mapping of SpoIIIL (in red) and Spo0A_C as a marker of sporulation (in green) on a maximum likelihood reference phylogeny of the *Firmicutes.* The mapping is based on a supermatrix containing 497 taxa with 3,776 amino acid positions and inferred with IQ-Tree using the evolutionary model LG + I + G4. Black circles correspond to ultrafast bootstrap supports higher than 80%, and the scale bar corresponds to the average number of substitutions per site. (B) Co-occurrence of SpoIIIL (in red) and ComG operon (in green). Co-occurrence of SpoIIIL and ComG in *B. subtilis* in highlighted in pale orange. Refer to high-resolution figure for finer details (https://doi.org/10.6084/m9.figshare.28937915.v1).

Previous work suggests that *spoIIIL* may be restricted to a subset of spore-forming Firmicutes [17,39,40]. To comprehensively gauge the phylogenetic distribution of *spoIIIL*, we conducted an analysis on 497 *Firmicutes* genomes (Fig 7A). This analysis revealed that *spoIIIL* is exclusively found in a subset of species within the *Bacillales* (Fig 7A), indicating that SpoIIIL function during sporulation reflects a species-specific specialization. We also examined the co-occurrence of *spoIIIL* with the *comG* operon in species of *Bacillales* where *spoIIIL* is present (Fig 7B). As mentioned in the introduction, in *B. subtilis spoIIIL* is the last gene in the *comG* operon and is transcribed with the *comG* genes by ComK. Interestingly, this does not appear to be the case in other species of *Bacillales.* Only a few species display the same genetic organization for *comG* and *spoIIIL* as that observed in *B. subtilis.* Other species exhibit different genetic organization, for example: *comG* appears to be transcribed in the opposite orientation to *spoIIIL* or *comG* is not found within the same genetic neighborhood as *spoIIIL*.

Based on the above we tested if SpoIIIL is required for DNA uptake during competence. We compared DNA uptake (i.e., transformation efficiency) by transforming WT and the *ΔspoIIIL* mutant with genomic DNA conferring spectinomycin resistance and counting the number of spectinomycin resistant colonies. The Δ*spoIIIL* mutant exhibited a 1.7-fold reduction in transformation efficiency compared to WT (S5C Fig), indicating that SpoIIIL is required for efficient DNA uptake. If so, the Δ*spoIIIL* mutant would likely exhibit a synergistic defect with other mutants defective in DNA uptake. To test this, we combined Δ*spoIIIL* with a mutant lacking *comFB* and *comFC (*Δ*spoIIIL* Δ*comFBC),* which encode two proteins that contribute to efficient DNA uptake [41]. While the Δ*comFBC* double mutant resulted in 7.9-fold reduction in transformation efficiency, the Δ*spoIIIL* Δ*comFBC* triple mutant resulted in 47.2-fold reduction (S5C Fig). Thus, SpoIIIL is required for efficient DNA uptake and likely functions with other ComG proteins in genetic competence in *B. subtilis*. Collectively, this data suggest that SpoIIIL contributes to genetic competence and sporulation in some *Bacillales* species.

## Discussion

**Fig 8:**
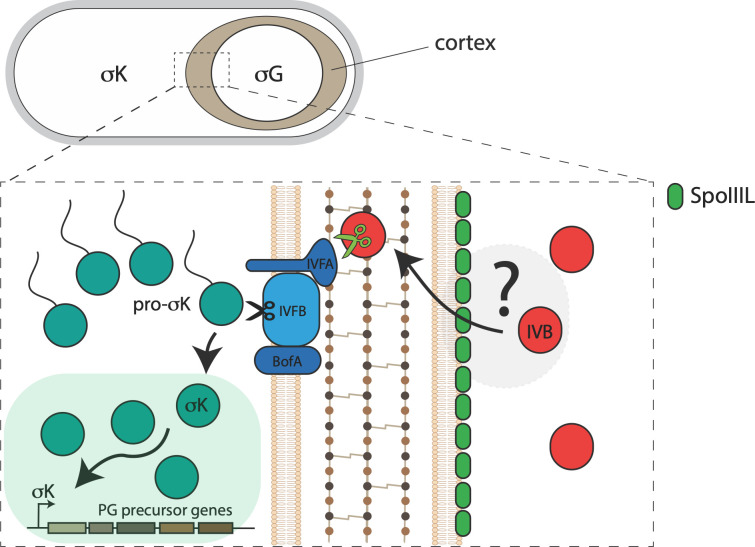
Diagrammatic model for the role of SpoIIIL in cell-cell signalling during the intermediate stages of sporulation. SpoIIIL (in green) influences the activity of SpoIVB (in red) by a yet-to-be-defined mechanism (grey area with question mark) that may involve changes to membrane fluidity and the ability of SpoIVB to undergo autoproteolysis. Fully active SpoIVB (green scissors) then initiates the RIP signalling cascade by cleaving SpoIVFA, which in turn activates the SpoIVFB intermembrane protease (IVFB, black scissors). Active SpoIVFB then cleaves inactive pro-σK to active σK. Active σK results in late mother cell transcription, which includes up-regulation of PG precursor genes required for cortex synthesis (brown area around in the forespore). For simplicity CtpB and BofC are not shown.

Here, we have redefined SpoIIIL’s function during sporulation. Our data indicate that SpoIIIL does not function in the A-Q complex. Instead, our data suggest that SpoIIIL is an additional forespore factor required for efficient signalling of σK activation in the mother cell (Fig 8). Thus, by redefining how SpoIIIL functions during sporulation, our findings rectify our understanding of the A-Q complex and provide additional insight into the RIP signalling pathway that activates late mother cell transcription.

Although SpoIIIL was initially suggested to function in the A-Q complex [10], our data indicate it does not. We found that unlike the A-Q complex proteins SpoIIQ, SpoIIIAH, SpoIIIAG and GerM that become enriched in the septal membranes during engulfment [15,18,25,26,42], SpoIIIL is initially uniformly localized in the forespore inner membrane before localizing in the forespore cytoplasm (Fig 2). Furthermore, while our data suggest that SpoIIIL contributes to efficient timing of σG activation, it is not required for σG activation *per se* (Fig 1). These observations are inconsistent with the idea that SpoIIIL functions in the A-Q complex. The slight, but reproducible delay in σG activation in the absence of SpoIIIL is interesting and suggests it could also function in the regulation of σG activation. Consistent with this idea, our data also suggest that σG activity remains high for a prolonged period of time in the absence of SpoIIIL (Fig 1). Deciphering the basis of these timing defects remains a challenge for future work.

While it is possible that changes in the timing of σG activation observed in the absence of SpoIIIL affect *spoIVB* transcription, and consequently delay σK processing, our analysis of *spoIVB* transcription in the absence of SpoIIIL suggests that this is unlikely to be the case (S3 Fig). Thus, collectively, our cytological, genetic and biochemical data favour the hypothesis that SpoIIIL functions in the RIP signalling pathway that leads to σK activation (Fig 8), and that SpoIIIL is the fourth forespore component of this signalling pathway in *B. subtilis*, in addition to SpoIVB, CtpB and BofC [13]. While the exact role of SpoIIIL in this pathway remains unclear, we hypothesize that SpoIIIL may function directly or indirectly as an activator of SpoIVB, since SpoIVB autoproteolysis is delayed and less efficient in the absence of SpoIIIL, which in turn affects the timing and efficiency of σK activation (Fig 5) and σK-dependent PG precursor accumulation required for efficient cortex synthesis (Fig 4). Another possibility is that SpoIIIL facilitates the secretion of SpoIVB into the intramembrane space, potentially functioning directly or indirectly as a co-factor of SpoIVB. Indeed SpoIIIL is well placed to fulfill this role, since it is associated with the inner forespore membrane around the same time that SpoIVB is made and secreted into the intermembrane space where it becomes active (Fig 2). Alternatively, by binding to the forespore membrane, SpoIIIL may simply alter the local lipid environment (e.g., fluidity) which could impact SpoIVB activity after it is secreted into the intermembrane space. Previous work has shown that the inactive, full-length form of SpoIVB is membrane associated, while the active, cleaved forms of SpoIVB are not [33]. Thus, SpoIIIL may change the lipid environment to promote SpoIVB activity and its subsequent dissociation from the membrane. Testing this model will require biochemical and biophysical experiments involving purified components. Experiments aimed at purifying recombinant full-length SpoIIIL have thus far been unsuccessful.

The change in SpoIIIL localization and its dependency on the cortex formation is intriguing (Fig 6). Cortex synthesis by the SpoVD and SpoVE ultimately depends on up-regulation of PG precursors genes by σK [9]. Since the absence of these cortex synthases is not known to affect σK activation or prevent the accumulation of σK-dependent PG precursors [9], the change in SpoIIIL localization from the membrane to the cytoplasm is unlikely to be a requirement for its function in the RIP signaling pathway leading to σK activation. Protein topology prediction tools and our computational simulations of SpoIIIL interacting with a lipid bilayer suggest that it associates with the membrane without spanning it (S1D Fig). Consistent with this idea, GFP-SpoIIIL and SpoIIIL-GFP display similar membrane localization patterns during early stages of development (Figs 2A and S1E). At later stages of sporulation, the change in SpoIIIL localization, and its dependency on cortex synthesis, may simply reflect a change in membrane fluidity that occurs because of cortex deposition, expansion of the intermembrane space and concurrent spore core dehydration, as suggested previously [43]. Indeed, previous work suggests that a reduction in membrane fluidity can result in membrane dissociation of peripheral membrane proteins [44]. An interesting question arising from the change in SpoIIIL localization, from membrane association to the cytoplasmic localization, is whether this reflects distinct roles during sporulation.

The genetic relationship between *ssdC* and *spoIIIL* identified by Tn-seq is interesting since both proteins are required for spore shape and cortex assembly [23]. Unlike the Δ*spoIIIL* Δ*murAB* double mutant which shows a large reduction in the number of phase bright spores but has a less severe reduction in heat-resistant spores, the Δ*spoIIIL* Δ*ssdC* double mutant retains the ability to produce many phase-bright spores that are however more susceptible to heat (Figs 3B and S2A). Our previous work suggests that the shape defect of the Δ*ssdC* mutant arises because of alterations in the cortex, which in turn leads to a heat-resistance and germination defect [23]. In the absence of SpoIIIL, cortex synthesis may also be affected by the reduction in PG precursors (Fig 4B). Although electron micrographs do not show major differences in cortex appearance between WT and the Δ*spoIIIL* mutant (Figs 3F and S5A), muropeptide analysis on mature spores suggests that the total amount of PG could be lower in the Δ*spoIIIL* mutant (S5D and S5E Fig). Interestingly, we also found that the Δ*spoIIIL* mutant spores germinate less efficiently (S5B Fig). Thus, while it remains unclear how SsdC contributes to the cortex [23], it is likely that the Δ*spoIIIL* Δ*ssdC* double mutant sporulation defect results from a combination of defects in the cortex that affect heat-resistance and germination. Deciphering the PG composition of mature spores lacking SsdC may reveal how this proteins affect the cortex and the underlying reason of its genetic relationship with SpoIIIL.

The narrow taxonomic distribution of SpoIIIL (Fig 7A) suggests that it has evolved to satisfy a unique requirement in the RIP cell-cell signalling pathway leading to σK activation in a subset of species within the *Bacillales*. However, it is unclear if *spoIIIL* was initially a sporulation gene before being incorporated into the *comG* operon, or if *spoIIIL* was originally part of the *comG* operon and later acquired a sporulation-specific promoter. Nonetheless, given that SpoIIIL co-occurs with the *comG* operon in many species of *Bacillales* (Fig 7B), it seems likely that it functions in both processes in many species. In this context, SpoIIIL may function as a moonlighting protein, executing distinct functions during different stages of development. Interestingly, previous work suggests an overlap between the genetic networks governing competence and sporulation [45–47], which may have driven the evolution of proteins that function in both developmental pathways. Future investigations into SpoIIIL’s role in competence may provide valuable insights into its function in the RIP signalling pathway that regulates late mother cell transcription, or vice-versa.

## Materials and methods

### General methods

*Bacillus subtilis* strains are listed in S2 Table, plasmids (and their construction details) are listed in S3 Table and oligonucleotide primers are listed in S4 Table. Sporulation induction was achieved through resuspension at 37°C following the Sterlini-Mandelstam method [48] or by nutrient depletion in a supplemented Difco sporulation medium (DSM) [49] consisting of 8 g/L Bacto nutrient broth (Difco), 0.1% (wt/vol) KCl, 1 mM MgSO_4_, 0.5 mM NaOH, 1 mM Ca(NO_3_)_2_, 0.01 mM MnCl_2_, and 0.001 mM FeSO_4_. Sporulation efficiency was determined through the heat-kill assay conducted on cultures grown for 30 hours at 37°C in the DSM medium. The total number of heat- resistant (80°C for 20 minutes) CFUs (colony-forming units) is compared with wild-type heat-resistant CFUs. Transformation efficiency was determined through a transformation assay conducted on cultures grown for 4 h at 37°C in 1XMC medium, followed by growth for 2 h at 37°C in the same spent medium but with added purified genomic DNA conferring spectinomycin resistance (0.01 mg; *ycgO::spec*).

### Transposon insertion sequencing

Transposon insertion sequencing (Tn-seq) was performed on wild-type and Δ*spoIIIL* libraries as described previously [10]. Approximately 750,000 transformants were pooled, aliquoted, and frozen. An aliquot was thawed, washed in DSM, and diluted into 50 mL DSM at an optical density at 600 nm (OD_600_) of 0.05. Samples were harvested 24 h later (T24). The T24 samples were incubated at 80°C for 20 min and then plated on LB agar. Approximately 750,000 colonies from germinated spores from each sample were pooled. Genomic DNA was extracted from both samples and digested with MmeI, followed by adapter ligation. Transposon-chromosome junctions were amplified in 16 PCR cycles. PCR products were gel purified and sequenced on the Illumina HiSeq platform using TruSeq reagents (Tufts University TUCF Genomics Facility). Reads were mapped to the *B. subtilis* 168 genome (NCBI NC_000964.3) and tallied at each TA site, and genes in which reads were statistically underrepresented or overrepresented were identified using the Mann-Whitney U test (full list can be found here: https://doi.org/10.6084/m9.figshare.28633853.v4). Visual inspection of transposon insertion profiles was performed with the Sanger Artemis Genome Browser and Annotation tool.

### Fluorescence microscopy

Live-cell fluorescence imaging was performed by placing cells on a 2% (w/v) agarose pad prepared with resuspension medium and set using a Gene Frame (Thermofisher). For sporulating cell cultures prepared using the resuspension method, at specific time points, 250 μL of culture was pelleted via centrifugation and resuspended in 10 μL of resuspension medium containing 0.05 mM TMA-DPH [1-(4-trimethylammoniumphenyl)-6-phenyl-1,3,5-hexatriene p-toluenesulfonate] or DAPI (10 µg/mL). Next, 2 μL of cell suspension was spread onto the agarose pad, and a coverslip was placed on top of the adhesive Gene Frame. Cells were imaged using a Zeiss Axio Observer 7 microscope equipped with a Plan-APOCHROMAT 100x/1.4 Oil Ph3 objective and a Colibri 7 Type R[G/Y]CBV-UV fluorescent light source. Images were captured with an Axiocam 712 mono camera. The TMA-DPH membrane dye was excited with a Zeiss 02 DAPI filter, using an exposure time of 100 ms. GFP was excited with a Zeiss 38 HE filter, with an exposure time of 150 ms. YFP and CFP was excited with a Zeiss 108HE filter, with an exposure time 150 and 300 ms, respectively. The mCherry fluorophore was excited with a Zeiss 90 HE LED filter, with an exposure time of 1000 ms. DAPI was excited with a Zeiss 02 DAPI filter, with an exposure time of 150 ms.

### Image analysis & Statistics

Microscopy images were processed by adjusting the brightness and contrast using the Fiji software. The MicrobeJ plugin [50], designed for the Fiji software [51], was used to analyze forespore shape (P_*spoIIQ-*_*cfp or P*_*spoIIQ*_*-mCherry*), σG activity (*P*_*sspB*_*-yfp)* and *spoIVB* transcription (P_*spoIVB*_-*cfp*). The proportion of cells containing phase-bright cortex (Fig 3E) or bright patches of pro-σK-CFP signal (Figs 5E and S4E) was determined manually, using the Cell Counter plug-in for Fiji.

To perform quantitative analysis of forespore shape and σG activity using P_*spoIIQ-*_*cfp* and *P*_*sspB*_*-yfp* signal, image background was first subtracted (Process > Subtract Background) to avoid false-positive detection of the fluorescent signal. Next, the “Bacteria” tab on MicrobeJ was set to “Smoothed” to detect the outline of CFP signal from the forespores in Channel 1 and the YFP signal from the same forespores in Channel 2. Three parameters: “Exclude on Edges”, “Shape descriptors”, “Segmentation” and “Intensity” were checked. The generated CFP outlines were further refined by setting the shape descriptors (area, length, width) for each time-point corresponding to the outlines of individual forespores. The manual editing tool was also used to resolve unprocessed forespores. Results of the analyses of Channel 1 and Channel 2, including aspect ratio and mean intensity were exported to Microsoft Excel to process raw data into line charts. A similar approach was used to analyze forespore shape in the SpoIIIL-GFP strain, in this case using P_*spoIIQ-*_*mCherry* set to Channel 1, to detect forespore outlines (no Channel 2 was used).

To perform quantitative analysis of *spoIVB* transcription using *P*_*spoIIQ*_*-mCherry* and P_*spoIVB*_-*cfp* signal, image background was first subtracted (Process > Subtract Background) to avoid false-positive detection of the fluorescent signal. Next, the “Bacteria” tab on MicrobeJ was set to “Smoothed” to detect the outline of mCherry signal from the forespores in Channel 1 and the CFP signal from the same forespores in Channel 2. Three parameters: “Exclude on Edges”, “Shape descriptors”, “Segmentation” and “Intensity” were checked. The generated mCherry outlines were further refined by setting the shape descriptors (area, length, width) for each time-point corresponding to the outlines of individual forespores. The manual editing tool was also used to resolve unprocessed forespores. Results of the analyses of Channel 2 (CFP), including mean intensity were exported to Microsoft Excel.

Plots were generated through inputting MicrobeJ data into Superplots (https://huygens.science.uva.nl/SuperPlotsOfData/) [52]. The non-parametric Kolmogorov-Smirnov test was used to compare the aspect ratio distribution, and P_*spoIVB*_-*cfp* or pro-σK-CFP signal distributions between populations of wild-type and mutant sporulating cells. ANOVA, followed by the Tukey’s multiple comparisons test were used to compare means between WT and mutant strains as indicated in the legend. Numerical values used in graphs can be found here: https://doi.org/10.6084/m9.figshare.28633853.v4.

### Immunoblot analysis

Whole-cell lysates from sporulating cells were prepared as previously described [23]. Samples were heated for 15 min at 50 °C prior to loading. Equivalent loading was based on the OD_600_ at T2.5, since after T3.5 optical density readings do not accurately correlate with cell density, due to changes in spore refractivity as a result of spore maturation. Samples were separated on a 10% polyacrylamide gel and transferred to a PVDF membrane. Membranes were blocked in 5% non-fat milk with 0.5% Tween-20 for 1 h. Blocked membranes were probed with anti-GFP (1:2500) (Abcam) or anti-SpoIVB (1:10000) primary antibodies diluted into PBS with 0.05% Tween-20 containing 3% Bovine Serum Albumin (w/v) (Sigma) at 4 °C overnight. Primary antibodies were detected with horseradish-peroxidase anti-rabbit antibodies (BioRad) and detected with ECL Prime Western Blotting Detection reagent (Amersham) as described by the manufacturer.

### PG precursor analysis

Sporulating cells (10 mL) were harvested at T4.5, normalized to an OD_600_ equal to 1 (measured at T3.5) and pelleted by centrifugation. Cell pellets were washed three times with ice-cold 0.9% NaCl, resuspended in 100 μL 0.9% NaCl, and boiled for 5 min to lyse the cells and extract the soluble peptidoglycan (PG) precursors. Lysates were centrifuged at 21,000 × *g* for 5 min to pellet insoluble material, and the supernatant was filtered (0.22 μm pore size) for LC-MS analysis. Extracted precursors were analysed using a UPLC system (Waters) with an Acquity UPLC BEH C18 column (130-Å, 1.7-μm, 2.1 × 150 mm) coupled to a Xevo G2-XS QTOF mass spectrometer (Waters). Separation was achieved using a gradient from 0.1% formic acid in water to 0.1% formic acid in acetonitrile over 18 min at 45°C. The QTOF operated in positive-ion mode with the MSe mode used for data acquisition. MS parameters were set as follows: capillary voltage, 3 kV; source temp, 120°C; desolvation temp, 350°C; sample cone voltage, 40 V; cone gas flow, 100 L/h; desolvation gas flow, 500 L/h. Data were acquired and processed using Masslynx software. Quantification was done by extracting ion chromatograms for the expected *m/z* ratios of each ion and integrating the resulting peaks. The extracted *m/z* values were: UDP-MurNAc: 702.09; UDP-M2:880.18; UDP-M4:1123.30 and UDP-M5: 1194.33.

### Muropeptide analysis of mature spores

Spore pellets of the WT and Δ*spoIIIL* mutant, normalized to an equal OD_600_, were resuspended in 2 mL decoating solution (50 mM Tris-HCl pH 8.0, 8 M urea, 1% SDS and 50 mM dithiothreitol) and incubated at 37 °C for 1 h with shaking. The spores were pelleted by centrifugation at 14,000 × *g* for 8 min and washed 5 times in 2 mL water to remove the SDS. The final pellets were resuspended in 0.9 mL trypsin digestion solution (20 mM Tris-HCl pH 8.0, 10 mM CaCl_2_, 0.1 mg/mL trypsin) and incubated overnight at 37 °C with shaking. The next day, 100 μL 10% SDS was added to each reaction, which were then boiled for 15 min. Insoluble PG sacculi were pelleted by ultracentrifugation at 150,000 × *g* for 13 min, washed 3 times with 3 mL water and resuspended in 100 μL water. Next, 10 μL of 2 mg/mL muramidase stock solution was added and the samples were incubated overnight at 37 °C with shaking to digest the PG into soluble muropeptides. The next day, the muramidase was inactivated by boiling for 5 min, and the samples were centrifuged at 24,000 × *g* for 15 min to remove the insoluble material. The supernatants were transferred to new tubes, dried in a SpeedVac system, and resuspended in 50 μL water. Next, 5 μL 0.5 M sodium borate buffer (pH 9) was added to adjust the pH to 8.5-9, before the muropeptides were reduced by adding 25 μL of freshly made 76 mg/mL NaBH_4_. The samples were incubated at RT for 20 min, and then adjusted to pH to 3.5-4 using 25% orthophosphoric acid. The final samples were filtered through a 0.22 μm pore-size filter for analysis by LC.

Liquid chromatography was carried out using an Acquity H-Class UPLC system (Waters) equipped with an Acquity UPLC BEH C18 column (2.1 mm × 150 mm, 130 Å pore size and 1.7 μm particle size, Waters) maintained at 45 °C. Chromatographic separation was achieved using a linear gradient from 0.1% formic acid in water to 0.1% formic acid in acetonitrile. UV detection was done at 204 nm. Peaks were identified using in-line LC-MS with a Xevo G2-XS mass spectrometer (Waters) operated in positive ion mode, with data collection performed in the data-independent MSe mode. MS parameters were set as follows: capillary voltage 3.0 kV, source temperature 120 °C, desolvation temperature 350 °C, sample cone voltage 40 V, cone gas flow 100 L h^−1^ and desolvation gas flow 500 L h^−1^. Data acquisition and processing was performed using the UNIFI software (Waters). Compounds were built and their expected masses calculated using the ChemSketch software (ACD labs).

UV chromatograms were integrated to quantify peaks using the Empower software (Waters). The relative molar abundance of each muropeptide was calculated by dividing peak areas by the calculated average molar mass of each compound relative to GlcNAc-MurNAc-tetrapeptide, then dividing the adjusted peak area by the total peak area to give percent molar abundance. Crosslinking was calculated as the percentage of peptide stems in crosslinks, and muramic δ-lactam was calculated as the percentage of total muramic acid residues.

### Chemical fixation, thin sectioning and electron microscopy of *B. subtilis* sporangia and spores

For TEM of mature spores, spore pellets were fixed in 3% (w/v) glutaraldehyde (prepared in 0.1M sodium cacodylate buffer) at 4°C overnight. Samples were washed in 0.1 M sodium cacodylate buffer and further incubated for 2 h at room temperature in 1% (w/v) osmium tetroxide for secondary fixation. Spore pellets were washed twice with 0.1 M sodium cacodylate buffer and dehydrated by incubating with increasing concentrations of ethanol [50% (v/v), 75% (v/v), 95% (v/v), 100% (v/v) dried ethanol], followed by two pure propylene oxide 15 min incubations for complete dehydration. Samples were incubated overnight at room temperature in a 1:1 mix of propylene oxide and Epon resin to allow for infiltration. Resin was removed and excess propylene oxide evaporated at room temperature. Samples were incubated for two consecutive 4h periods in pure Epon resin before being embedded into the final fresh resin. Resin was polymerized by incubation at 60°C for 48 h. Thin sections (80 nm) were produced using an Ultracut E Ultramicrotome (Reichert-Jung) and floated onto 300-square mesh nickel TEM grids. Sections were stained in 3% (w/v) uranyl acetate for 30 min, washed with dH_2_O, stained with Reynold’s lead citrate for 5 min and further washed with dH_2_O. Sections were imaged on a Tecnai G2 spirit BioTwin (FEI) 120 kV microscope, at a magnifications between 16,000–32,000 x (pixel size of 0.56 and 0.28 nm), equipped with an Orius SC1000B CCD camera (Gatan).

For TEM of sporangia, sporulating cells were harvested at T9 by centrifugation at 3,220 × *g* for 10 min. The resulting cell pellet (~ 1.5 μL) was placed on the 200-μm side of a type A 3-mm gold platelet (Leica Microsystems), covered with the flat side of a type B 3-mm aluminum platelet (Leica Microsystems), and vitrified by high-pressure freezing using an HPM100 system (Leica Microsystems). Freeze-substitution was carried out at −90°C for 80 h in acetone supplemented with 1% OsO_4_. The samples were then gradually warmed at a rate of 2°C/h to −60°C using an AFS2 system (Leica Microsystems). After incubation at −60°C for 8–12 h, the temperature was raised to −30°C (2°C/h), and then to 0 °C within 1 h to enhance the osmium action and improve the membrane contrast. The sample was then cooled down to −30 °C within 30 min and subsequently rinsed 4 times in pure acetone. For resin embedding, the samples were progressively infiltrated with increasing concentrations of Embed812 resin (EMS) in acetone (1:2, 1:1, 2:1 [v/v]) over 2 h for each ratio, while gradually raising the temperature to 20°C. Pure resin was then added at room temperature, and polymerization was carried out at 60°C for 48 h. Thin sections (80 nm) were prepared using an ultramicrotome UC7 (Leica Microsystems) and collected on formvar-carbon-coated 100-mesh copper grids (Agar Scientific). The sections were post-stained with 2% aqueous uranyl acetate for 5 min, rinsed in water, further stained with lead citrate for 2 min and rinsed again. The samples were imaged using a Tecnai G2 spirit BioTwin (FEI) 120 kV microscope, at a magnifications between 16,000–32,000 x (pixel size of 0.56 and 0.28 nm), equipped with an Orius SC1000B CCD camera (Gatan).

### Phylogenetic and co-conservation analysis

We assembled a local database of *Firmicutes* containing 497 genomes and inferred the corresponding reference phylogenetic tree as described in Chan *et al.,* 2022 [28]. To infer sporulating taxa in the Firmicutes, we searched for homologues of Spo0A against our Firmicutes database using the pfam domain PF08769 (Spo0A_C) and the HMMsearch tool from the HMMER package [53], with the option cut_ga. To infer the presence of SpoIIIL, we used the pfam domain PF14038 (YqzE) and HMMsearch tool. All SpoIIIL hits were retrieved and curated using alignment, phylogeny and domains to discard false positives. The presence of Spo0A and SpoIIIL was then mapped on the reference phylogeny of the Firmicutes using IToL [54].

To explore the co-conservation of SpoIIIL with the ComG operon in the Firmicutes, we retrieved the pfam domains PF00437, PF11773, PF15980, and PF14173 corresponding to the proteins ComGA, ComGE, ComGF, and ComGG respectively. As the remaining proteins do not have a corresponding pfam domain, we extracted the alignments corresponding to NF041012 (T4P_ComGB) and NF040982 (ComGD) NCBI families, and the COG family COG4537 (ComGC) from the Conserved Domain Database [55]. HMM profiles were first generated using the downloaded alignments with HMMbuild tool and then searched against the *Firmicutes* database using HMMsearch. All the hits retrieved were used to highlight the occurrence of one or more proteins from the ComG operon next to SpoIIIL using GeneSpy [56].

### Computational simulations

An AlphaFold2 [57] prediction of the full length SpoIIIL protein, was inserted along its hydrophobic facing residues onto a 20:70:10 POPG:POPE:CDL1 membrane using the MemProtMD pipeline [58]. After automatic partial submersion in the lipids as predicted by Memembed [59] and stabilization by coarse Grained simulation under default MemProtMD parameters, CG2AT [60] was used to produce a full atom simulation box for production simulation of the SpoIIIL-membrane assembly. The full atom box was then simulated using GROMACs [61] charmm36 force fields for 500 ns at 310K in 150mM NaCl at pH 7 (S1 Movie).

## Supporting information

S1 Movie Computational simulation of SpoIIIL on a lipid bilayer. (MOV)

S1 Table List of Top Tn-seq hits validated by Heat-Kill assay. (DOCX)

S2 Table *Bacillus subtilis* strains used in this study. (DOCX)

S3 Table Plasmids used in this study. (DOCX)

S4 Table Oligonucleotide primers used in this study. (DOCX)

S1 Fig Forespore shape data, validation of SpoIIIL fluorescent fusions and membrane nanoscale simulation of SpoIIIL. **(A)** Forespore aspect ratio in WT and ∆*spoIIIL* at 5 hours after the onset of sporulation (data representative of 3 biological replicates, each shown by a different colour; **** *p* < 0.0001, Kolmogorov-Smirnov test). **(B)** Sporulation efficiency of SpoIIIL-GFP and GFP-SpoIIIL expressed *in trans*, as the sole source of SpoIIIL (n = 3, mean ± STDEV). **(C)** Forespore aspect ratio of SpoIIIL-GFP expressed *in trans*, as the sole source of SpoIIIL, at 5.5 hours after the onset of sporulation (data representative of 3 biological replicates, each shown by a different colour). Representative images of the strain used in the analysis of forespore shape are shown to the left. P_*spoIIQ-*_*mCherry* was used to identify forespores for quantitative image analysis of forespore shape. Scale bar is 2 µm. **(D)** SpoIIIL structure and computational simulation of SpoIIIL. (i) SpoIIIL AlphaFold 2 structure showing its three alpha helices and amino acid side chains; N-terminus (N) is shown to the left and C-terminus (C) to the right. The protein is labelled in a Kyte-Doolittle scale of blue and yellow: yellow corresponds to hydrophobic residues and blue, hydrophilic residues. (ii & iii) SpoIIIL remains membrane associated during computational simulation (500 ns) with a lipid bilayer; (ii) shows a side view and (iii) a bottom view. Refer to S1 Movie for full simulation (https://doi.org/10.6084/m9.figshare.28609064.v1). **(E)** Representative images of GFP-SpoIIIL at T3.5 and T5.5. GFP-SpoIIIL is shown in cyan and membrane in red in the merge. Scale bar is 2 µm. **(F)** Representative images of MalF-GFP expressed in the forepore under σF control (P_*spoIIQ*_-*malF-gfp*) at T3 and T5. Fewer sporangia produce MalF-GFP at T5 due to the temporal regulation of σF. MalF-GFP is shown in cyan and membrane in red in the merge. Scale bar is 2 µm.
(TIF)

S2 Fig Spore refractivity, spore shape, σG-activity and validation of various mutant strains. **(A)** Representative phase contrast images showing spore refractivity at 30 hours after the onset of sporulation in the WT and in ∆*spoIIIL,* ∆*ssdC* and ∆*spoIIIL* ∆*ssdC* mutant sporangia. Scale bar is 2 μm **(B)** Mean sporulation efficiency (% spores relative to WT; n = 3, ± STDEV) showing complementation of the ∆*spoIIIL* ∆*murAB* double mutant defect by reintroduction of *murAB* at its native locus (+MurAB). **(C)** Mean intensity (± STDEV of 3 biological replicates; n > 1000, for each biological replicate) of σG-dependent YFP signal in WT, ∆*spoIIIL,* ∆*ssdC* and ∆*spoIIIL* ∆*ssdC* mutant forespores during a sporulation time-course. **(D)** Mean aspect ratio (± STDEV of 3 biological replicates; n > 500, for each biological replicate) of WT and ∆*spoIIIL,* ∆*ssdC* and ∆*spoIIIL* ∆*ssdC* mutant forespores during a sporulation time-course. **(E)** Representative images of ∆*ssdC* and ∆*spoIIIL* ∆*ssdC* mutant sporangia expressing CFP (under the control of σF-dependent promoter) and YFP (under the control of a control of σG-dependent promoter) at T3.5 and T5. Merged images show membrane in red and YFP in cyan. Scale bar is 2 μm. **(F)** Mean intensity (± STDEV of 3 biological replicates; n > 1000, for each biological replicate) of σG-dependent YFP signal in WT and ∆*spoIIIL,* ∆*murAB* and ∆*spoIIIL* ∆*murAB* mutant forespores, during a sporulation time-course. **(G)** Mean aspect ratio (± STDEV of 3 biological replicates; n > 500, for each biological replicate) of WT and ∆*spoIIIL,* ∆*murAB* and ∆*spoIIIL* ∆*murAB* mutant forespores, during a sporulation time-course. **(H)** Representative images of ∆*murAB* and ∆*spoIIIL* ∆*murAB* mutant sporangia expressing CFP (under the control of σF-dependent promoter) and YFP (under the control of a control of σG-dependent promoter) at T3.5 and T5. Merged images show membrane in red and YFP in cyan. Scale bar is 2 μm.
(TIF)

S3 Fig Pro-σK-CFP localization, immunoblots and P_*spoIVB*_ transcriptional-reporter data. **(A)** Representative images of pro-σK-CFP localization at 4.5 (T4.5) hours after the onset of sporulation in WT and ∆*spoIVB.* Merge shows DNA stained with DAPI in red and pro-σK-CFP in cyan. Scale bar is 2 μm. **(B)** Uncropped Immunoblots comparing SpoIVB levels in WT and various mutants. Full-length (FL) and autoproteolyzed (AP) forms of SpoIVB are pointed out on the right and the position of molecular weight markers are shown to the left. **(C)** Representative images of P_*spoIVB-*_*cfp* reporter at 4.5 (T4.5) and 5.5 (T5.5) hours after the onset of sporulation in WT and ∆*spoIIIL,* ∆*murAB,* ∆*spoIIIL* ∆*murAB* mutant backgrounds. P_*spoIIQ-*_*mCherry* was used to identify forespores for quantitative image analysis of P_*spoIVB-*_*cfp* signal. Scale bar is 2 μm. **(D)** Mean intensity (± STDEV of 3 biological replicates; n > 500, for each biological replicate) of P_*spoIVB*_-*cfp* transcriptional reporter signal in WT and ∆*spoIIIL,* ∆*murAB* and ∆*spoIIIL* ∆*murAB* mutant forespores, at T4.5 (left) and T5.5 (right). **(E)** Representative images of pro-σK-CFP localization at 3.5 (T3.5), 4.5 (T4.5) and 5.5 (T5.5) hours after the onset of sporulation in WT and ∆*spoIIIL,* ∆*murAB,* ∆*spoIIIL* ∆*murAB* and ∆*spoIVB* mutant backgrounds. Merged shows DNA stained with DAPI in red and pro-σK-CFP in cyan. Scale bar is 2 μm.
(TIF)

S4 Fig Sporulation efficiency and σ^K^ activation in the absence of SpoIIIL and CtpB (A) CtpB is a second forespore-produces protease that contributes to SpoIVFA cleavage and fine-tunes the σK activation RIP signalling pathway. **(B)** Tn-seq profiles at the *ctpB* locus of WT and ∆*spoIIIL* mutant cells following 30 h of growth and sporulation in exhaustion medium. The height of the vertical lines represents the number of sequencing reads at each position (scale is 200 sequencing reads). Shaded regions highlight the significant reduction in sequencing reads at the *ctpB* locus. **(C)** Representative images of pro-σK-CFP localization at 3.5 (T3.5), 4.5 (T4.5) and 5.5 (T5.5) hours after the onset of sporulation in WT and ∆*spoIIIL,* ∆*ctpB and* ∆*spoIIIL* ∆*ctpB* mutant backgrounds. Scale bar is 2 μm. **(D)** Sporulation efficiency (% spores relative to WT) of ∆*ctpB,* ∆*spoIIIL and* ∆*spoIIIL* ∆*ctpB* mutants (n = 3, mean ± STDEV; *p < 0.05, Tukey’s multiple comparisons test). **(E)** Mean frequency of processed pro-σK-CFP at 3.5, 4.5 and 5.5 hours after the onset of sporulation in WT and ∆*spoIIIL,* ∆*ctpB and* ∆*spoIIIL* ∆*ctpB* mutant backgrounds (errors bars are ± STDEV of 3 biological replicates, > 100 cells for each biological replicate; *p < 0.05, ***p < 0.001, ****p < 0.0001, Tukey’s multiple comparisons test).
(TIF)

S5 Fig Spore envelope, spore germination, spore PG and transformation efficiency of the WT and ∆*spoIIIL* mutant. **(A)** Representative electron micrographs of mature spore sections of the WT and and ∆*spoIIIL* mutant. The yellow line highlights the region occupied by the cortex and the blue line the region occupied by the coat. Scale bar is 100 nm. **(B)** Germination efficiency of WT (blue) and ∆*spoIIIL* mutant (red) spores. Purified, mature spores of each strain were inoculated into LB broth at 37ºC, with constant agitation (200 rpm) to an OD_600_ of 1.2 (100%) and OD changes were monitored overtime (n = 3, ± STDEV). The ∆*spoIIIL* mutant exhibits a delay in germination, as demonstrated by the less pronounced reduction in OD overtime. **(C)** Mean transformation efficiency (% relative to WT) of the ∆*spoIIIL,* ∆*comFBC* and ∆*spoIIIL* ∆*comFBC* mutants (n = 3, ± STDEV; *p < 0.05, *p < 0.0001, Tukey’s multiple comparisons test). **(D)** Spore PG in WT and ∆*spoIIIL* mutant. Left panels shows % muramic acid moieties present as muramic δ-lactam; middle panels shows % peptide stems in crosslinks and right panel shows relative PG amount obtained from spore samples, determined by total area under identified peaks. **(E)** UPLC UV chromatogram of muramidase-digested PG obtained from WT and ∆*spoIIIL* mutant spores, with identified peaks numbered (top panel); (n = 2, mean ± STDEV; ns - not statistically significant, Tukey’s multiple comparisons test). Proposed identities and structures of identified peaks determined by UPLC-MS (bottom panel).
(TIF)
