# Supplementary material for: SpoIIIL is a forespore factor required for efficient cell-cell signalling during Bacillus subtilis sporulation
Source: PLoS Genet. 2025 Jul 3;21(7):e1011768. doi: 10.1371/journal.pgen.1011768 (PMC12251134; doi:10.1371/journal.pgen.1011768)
Supplement: S1 Table — (DOCX) [file pgen.1011768.s002.docx]

**Table S1. List of Top Tn-seq hits validated by Heat-Kill assay**

| gene | *p*-value | Tn. fold change ∆*spoIIIL* /WT^a^ | possible function | % spores (±STDEV)  gene / ∆*spoIIIL*^b^ | spore fold-difference / ∆*spoIIIL*^c^ |
| --- | --- | --- | --- | --- | --- |
| *spoIIIL* | - | - | spore shape & efficient assembly of the spore cortex | 31.9 ± 4.5 | - |
| *pbpF* | 0.00001 | 8.8 | bifunctional glucosyltransferase/ transpeptidase, synthesis of spore peptidoglycan | 4.5 ± 1.9 | 7.1 |
| *spsD* | 0.0004 | 3.7 | legionaminic acid synthesis | 5.5 ± 3.7 | 12.6 |
| *ctpB* | <0.00001 | 11.8 | carboxy-terminal processing serine protease, cleaves SpoIVFA | 6.3 ± 1.7 | 12.1 |
| *ssdC* | 0.0009 | 250 | assembly of the spore cortex | 0.01 ± 0.05 | 3.2 x10^4^ |
| *murAB* | 0.00001 | 76.9 | peptidoglycan precursor biosynthesis | 0.38 ± 0.0002 | 83.9 |
| *safA* | 0.0003 | 10 | spore coat formation | 4.7 ± 0.2 | 9.3 |
| *cotE* | 0.0003 | 71.4 | assembly of the outer spore coat | 1.9 ± 0.7 | 36.1 |
| *spoVID* | 0.0043 | 21.8 | spore coat morphogenetic protein | 2.8 ± 0.07 | 8.7 |

**^a^** Fold-difference in the number of transposon insertions: e.g. the ∆*pbpF* mutant had 8.8-fold less transposon insertions in the Δ*spoIIIL* than the WT.

**^b^** Sporulation efficiency (% relative to WT) of double mutants: e.g. the ∆*spoIIIL* ∆*pbpF* double mutant produced 4.5% spores.

**^c^** Fold-difference in sporulation efficiency of the double mutant relative to the ∆*spoIIIL* mutant: e.g. the ∆*spoIIIL* ∆*pbpF* double mutant produced 7.1-fold fewer spores compared to the ∆*spoIIIL* mutant.
