# Supplementary material for: SpoIIIL is a forespore factor required for efficient cell-cell signalling during Bacillus subtilis sporulation
Source: PLoS Genet. 2025 Jul 3;21(7):e1011768. doi: 10.1371/journal.pgen.1011768 (PMC12251134; doi:10.1371/journal.pgen.1011768)
Supplement: S2 Table — (DOCX) [file pgen.1011768.s003.docx]

**Table S2: *Bacillus subtilis*** strains used in this study.

| **Strain** | **Genotype** | **Source** |
| --- | --- | --- |
| 168 | *auxotrophic wild-type strain* | Zeigler *et al.,* 2008 [4] |
| bCR1565 | *ssdC::lox72* | This work |
| bDMA001 | *spoIIIC-cfp (cat)* | This work |
| bDMA002 | *spoIIIl::erm, murAB::murAB* | This work |
| bDMA005 | *spoIIIC-cfp (cat), spoIIIL::erm* | This work |
| bDMA006 | *spoIIIL::erm, murAB::lox72* | This work |
| bDMA007 | *spoIIIC-cfp (cat), ctpB::kan* | This work |
| bDMA008 | *spoIIIC-cfp (cat), spoIIIL:erm, ctpB::kan* | This work |
| bDMA032 | *murAB::lox72, spoIIIC-cfp (cat)* | This work |
| bDMA033 | *spoIIIL::erm, murAB::lox72, spoIIIC-cfp (cat)* | This work |
| bDMA082 | *spoIIIL::erm* | This work |
| bDMA103 | *spoIVB::spec, spoIIIC-cfp (cat)* | This work |
| bDMA167 | *spoIIIL::erm, yhdG::PspoIIIL-spoIIIL-gfp(cat), ycgO::PspoIIQ-optRBS-mCherry (kan)* | This work |
| bDMA185 | *spoIVB::spec* | This work |
| bDMA214 | *sacA::PspoIVB-cfp (erm), ycgO::PspoIIQ-optRBS-mCherry (kan), murAB::lox72* | This work |
| bDMA215 | *sacA::PspoIVB-cfp (erm), ycgO::PspoIIQ-optRBS-mCherry (kan), murAB::lox72, spoIIIL::lox72* | This work |
| bHC293 | *murAB::murAB* | Chan *et al.,* 2022 [1] |
| bHC352 | *murAB::lox72* | Chan *et al.,* 2022 [1] |
| bPO012 | *pbpF::kan, spoIIIL::erm* | This work |
| bPO019 | *spsD::kan, spoIIIL::erm* | This work |
| bPO030 | *spoIIIL::erm* | This work |
| bPO032 | *spoIIIL::erm, ctpB::kan* | This work |
| bPO033 | *spoIIIL::erm, safA::kan* | This work |
| bPO034 | *spoIIIL::erm, ssdC::lox72* | This work |
| bPO035 | *murAB::erm, spoIIIL::lox72* | This work |
| bPO046 | *yhdG::PsspB-optRBS-yfp (tet), amyE::PspoIIQ-cfp (cat)* | This work |
| bPO083 | *yhdG::PspoIIIL-spoIIIL-sfgfp (cat), spoIIIL::erm* | This work |
| bPO099 | *murAB::erm, yhdG::PsspB-optRBS-yfp(tet), amyE::PspoIIQ-cfp (cat)* | This work |
| bPO138 | *spoIIIL::erm, yhdG::PsspB-optRBS-yfp(tet), amyE::PspoIIQ-cfp (cat), cotE::kan* | This work |
| bPO141 | *spoIIIL::erm, yhdG::PsspB-optRBS-yfp (tet), amyE::PspoIIQ-cfp (cat), spoVID::kan* | This work |
| bPO146 | *spoVD::spec, spoVE::kan, yhdG::PspoIIIL-spoIIIL-sfgfp(cat), spoIIIL::erm* | This work |
| bPO048 | *spoIIIL::erm, yhdG::PsspB-optRBS-yfp (tet), amyE::PspoIIQ-cfp (BS)(cat)* | This work |
| bPO096 | *ssdC::erm, yhdG::PsspB-optRBS-yfp (tet), amyE::PspoIIQ-cfp(BS)(cat)* | This work |
| bPO098 | *amyE::PspoIIQ-cfp(Bs) (cat), yhdG::PsspB-optRBS-yfp(tet), spoIIIL::erm, ssdC::lox72* | This work |
| bPO099 | *murAB::erm, yhdG::PsspB-optRBS-yfp(tet), amyE::PspoIIQ-cfp(BS)(cat)* | This work |
| bPO101 | *amyE::PspoIIQ-cfp(Bs)(cat), yhdG::PsspB-optRBS-yfp(tet), murAB::erm, spoIIIL::lox72* | This work |
| bPO215 | *yhdG::PspoIIIL-sfgfp-spoIIIL (cat), spoIIIL::erm* | This work |
| bKC144 | *sacA::PspoIVB-cfp (erm), ycgO::PspoIIQ-optRBS-mCherry (kan)* | This work |
| bKC146 | *sacA::PspoIVB-cfp (erm), ycgO::PspoIIQ-optRBS-mCherry (kan), spoIIIL::lox72* | This work |
| PY79 | *prototrophic wild-type strain* | Youngman *et al,* 1983 [2] |
| bCR1236 | *spoIIIL::erm* | Meeske *et al.,* 2016 [3] |
| bCR1238 | *comF(BC)::cat* | This work |
| bKC133 | *spoIIIL::erm, comF(BC)::cat* | This work |

**REFERENCES**

1. Chan H, Taib N, Gilmore MC, Mohamed AMT, Hanna K, Luhur J, et al. (2022) Genetic Screens Identify Additional Genes Implicated in Envelope Remodeling during the Engulfment Stage of *Bacillus subtilis* Sporulation. *mBio* 13(5):e0173222.

2. Youngman, P.J., Perkins, J.B., and Losick, R. (1983) Genetic transposition and insertional mutagenesis in *Bacillus subtilis* with *Streptococcus faecalis* transposon Tn917. *Proc Natl Acad Sci* U S A 80: 2305–2309.

3. Meeske AJ, Rodrigues CD, Brady J, Lim HC, Bernhardt TG, Rudner DZ. (2016) High-Throughput Genetic Screens Identify a Large and Diverse Collection of New Sporulation Genes in Bacillus subtilis. *PLoS Biol* 14(1):e1002341.

4. Zeigler DR, Prágai Z, Rodriguez S, Chevreux B, Muffler A, et al. (2008) The origins of 168, W23, and other *Bacillus subtilis* legacy strains. *J Bacteriol* 190: 6983–95. pmid:18723616
