# Supplementary material for: SpoIIIL is a forespore factor required for efficient cell-cell signalling during Bacillus subtilis sporulation
Source: PLoS Genet. 2025 Jul 3;21(7):e1011768. doi: 10.1371/journal.pgen.1011768 (PMC12251134; doi:10.1371/journal.pgen.1011768)
Supplement: S3 Table — (DOCX) [file pgen.1011768.s004.docx]

**Table S3: Plasmids** used in this study.

| ****Plasmid**** | Description | Source |
| --- | --- | --- |
| **pPO001** | *yhdG::PsspB-optRBS-yfp (phleo)* | This work |
| **pPO002** | *yhdG::PsspB-optRBS-yfp (tet)* | This work |
| **pPO003** | *yhdG::PspoIIIL-spoIIIL-sfgfp (cat)* | This work |
| **pPO008** | *yhdG::PspoIIIL-sfgfp-spoIIIL (cat)* | This work |
| **pDMA019** | *sacA::PspoIVB-RBS-cfp (erm)* | This work |

**Plasmid Construction**

**pDMA019 [*sacA::P_spoIVB_-optRBS-cfp* (*erm)*]** was generated in two-way ligation with a *Eco*RI-*Hind*III product containing the promoter of *spoIVB* (IDT gBlock) and pCR208 [*sacA::P_veg_-optRBS-cfp (erm)*] cut with *Eco*RI and *Hind*III.

**pPO001 [*yhdG::P_sspB_-optRBS-yfp (phleo)*]** was generated in a three-way ligation with an *Eco*RI-*Xho*I PCR product containing the *sspB* promoter region (oligonucleotide primers oPO19 & oPO20 and 168 genomic DNA as template), an *Xho*I-*Bam*HI PCR product containing *optRBS-yfp* (phleo) (oligonucleotide primers oPO21 & oAT093 and plasmid pJL009 (*sacA::P_spoIIQ_-optRBS-yfp (erm)*) as template), as well as pBB280 cut with *Eco*RI and *Bam*HI. pBB280 (*yhdG::phleo*) is an ectopic integration vector for double cross over integration at the non-essential *yhdG* locus (David Rudner laboratory stock).

**pPO002 [*yhdG::P_sspB_-optRBS-yfp (tet)*]** was generated in a two-way ligation with an *Eco*RI-*Bam*HI insert containing the *P_sspB_-optRBS-yfp* from pPO01 and pBB281 cut with *Eco*RI and *Bam*HI. pBB281 (*yhdG::tet*) is an ectopic integration vector for double cross over integration at the non-essential *yhdG* locus (David Rudner laboratory stock).

**pPO004 [*yhdG::P_spoIIIL_-spoIIIL-sfgfp (cat)]*** was generated in a two-way ligation with an *Eco*RI-*Xho*I PCR product containing the *spoIIIL* open-reading frame and promoter region, (oligonucleotide primers oPO25 & oPO26 and 168 genomic DNA as template) and pCR227 digested with *Eco*RI-*Xho*I. pCR227 [*yhdG-P_gerM_-gerM-sfgfp (cat)*] contains *sfgfp* vector and is an ectopic integration for double cross over integration at the non-essential *yhdG* locus (Christopher Rodrigues laboratory stock).

**pPO008 *[yhdG::P_spoIIIL_-sfgfp-spoIIIL(cat)]*** was generated in a three-way ligation with an *Eco*RI-*Xho*I PCR product containing *sfgfp* fused to the *spoIIIL* promoter (*P_spoIIIL_*-*sfgfp; (*oligonucleotide primers oPO26 & oPO56 and Gibson assembly product as template), a *Xho*I-*Bam*HI PCR product containing the *spoIIIL* open-reading frame (oligonucleotide primers oPO57 & oPO58 and 168 genomic DNA as template) and pCR227 digested with *Eco*RI-*Bam*HI. The *Eco*RI-*Xho*I PCR product containing P*_spoIIIL_*-*sfgfp* was initially a Gibson assembly product comprising of two pieces of DNA that were joined such that transcriptional and translation elements of *spoIIIL* are preserved when joined to *sfgfp* : (1) *Eco*RI-*PspoIIIL* (oligonucleotide primers oPO26 & oPO54 and 168 gDNA as template), and (2) *sfgfp*-*Xho*I (oligonucleotide primers oPO55 & oPO56 and pPO004 as template).
