# Supplementary material for: SpoIIIL is a forespore factor required for efficient cell-cell signalling during Bacillus subtilis sporulation
Source: PLoS Genet. 2025 Jul 3;21(7):e1011768. doi: 10.1371/journal.pgen.1011768 (PMC12251134; doi:10.1371/journal.pgen.1011768)
Supplement: S4 Table — (DOCX) [file pgen.1011768.s005.docx]

**Table S4: Oligonucleotide primers used in this study**.

| ****Oligonucleotide**** | ****Sequence***** |
| --- | --- |
| oPO019 | cgcGAATTCgatgttctgaaacaatgatacaatg |
| oPO020 | gcgCTCGAGtttttatttagtatggttgggttaactgg |
| oPO021 | gcgCTCGAGacataaggaggaactactatgagt |
| oPO025 | cgcCTCGAGttttttccgtttcagccaaagtcggaagcc |
| oPO026 | cgcGAATTCatcaacttaagagtgtcaacgg |
| oPO054 | ataatcattcgttttcacgtgatc |
| oPO055 | cgtgaaaacgaatgattattctaaaggtgaagaactgttc |
| oPO056 | cgcCTCGAGgccgcttgagcctccagatgatcctttgtagagctcatccatgccgtgcg |
| oPO057 | cgcCTCGAGgtgaaaacgaatgattatg |
| oPO058 | cgcGGATCCttattttttccgtttcagccaaagtcggaagcc |
| oAT093 | cgcGGATCCttatttgtatagttcatccatgcc |

^*capital letters indicate restriction sites^
