# Supplementary figures and images for: SpoIIIL is a forespore factor required for efficient cell-cell signalling during Bacillus subtilis sporulation
Source: PLoS Genet. 2025 Jul 3;21(7):e1011768. doi: 10.1371/journal.pgen.1011768 (PMC12251134; doi:10.1371/journal.pgen.1011768)

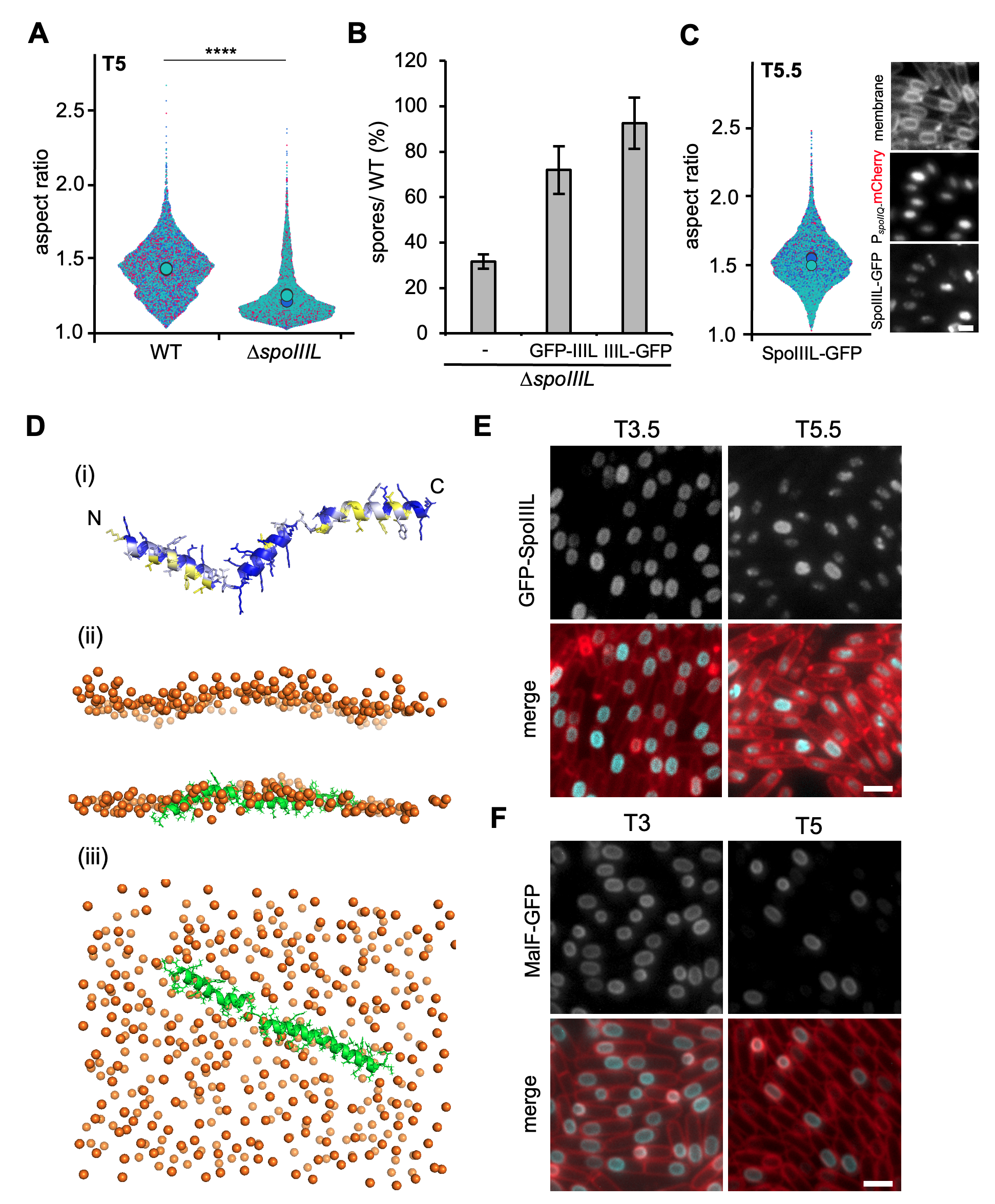

Supplement: S1 Fig — (A) Forespore aspect ratio in WT and ∆spoIIIL at 5 hours after the onset of sporulation (data representative of 3 biological replicates, each shown by a different colour; **** p < 0.0001, Kolmogorov-Smirnov test). (B) Sporulation efficiency of SpoIIIL-GFP and GFP-SpoIIIL expressed in trans, as the sole source of SpoIIIL (n = 3, mean ± STDEV). (C) Forespore aspect ratio of SpoIIIL-GFP expressed in trans, as the sole source of SpoIIIL, at 5.5 hours after the onset of sporulation (data representative of 3 biological replicates, each shown by a different colour). Representative images of the strain used in the analysis of forespore shape are shown to the left. PspoIIQ-mCherry was used to identify forespores for quantitative image analysis of forespore shape. Scale bar is 2 µm. (D) SpoIIIL structure and computational simulation of SpoIIIL. (i) SpoIIIL AlphaFold 2 structure showing its three alpha helices and amino acid side chains; N-terminus (N) is shown to the left and C-terminus (C) to the right. The protein is labelled in a Kyte-Doolittle scale of blue and yellow: yellow corresponds to hydrophobic residues and blue, hydrophilic residues. (ii & iii) SpoIIIL remains membrane associated during computational simulation (500 ns) with a lipid bilayer; (ii) shows a side view and (iii) a bottom view. Refer to S1 Movie for full simulation (https://doi.org/10.6084/m9.figshare.28609064.v1). (E) Representative images of GFP-SpoIIIL at T3.5 and T5.5. GFP-SpoIIIL is shown in cyan and membrane in red in the merge. Scale bar is 2 µm. (F) Representative images of MalF-GFP expressed in the forepore under σF control (PspoIIQ-malF-gfp) at T3 and T5. Fewer sporangia produce MalF-GFP at T5 due to the temporal regulation of σF. MalF-GFP is shown in cyan and membrane in red in the merge. Scale bar is 2 µm. (TIF) [file pgen.1011768.s006.tif]

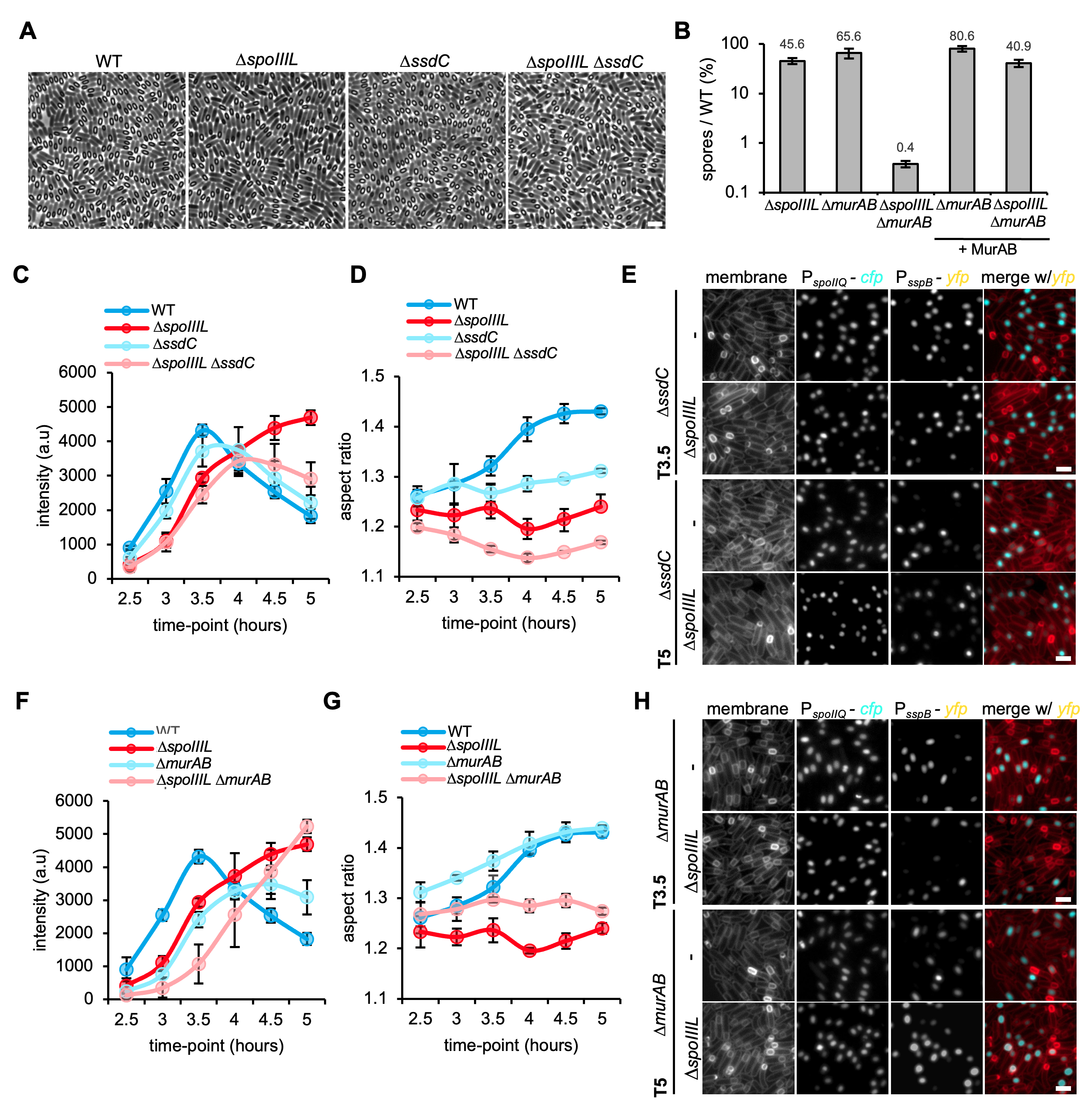

Supplement: S2 Fig — (A) Representative phase contrast images showing spore refractivity at 30 hours after the onset of sporulation in the WT and in ∆spoIIIL, ∆ssdC and ∆spoIIIL ∆ssdC mutant sporangia. Scale bar is 2 μm (B) Mean sporulation efficiency (% spores relative to WT; n = 3, ± STDEV) showing complementation of the ∆spoIIIL ∆murAB double mutant defect by reintroduction of murAB at its native locus (+MurAB). (C) Mean intensity (± STDEV of 3 biological replicates; n > 1000, for each biological replicate) of σG-dependent YFP signal in WT, ∆spoIIIL, ∆ssdC and ∆spoIIIL ∆ssdC mutant forespores during a sporulation time-course. (D) Mean aspect ratio (± STDEV of 3 biological replicates; n > 500, for each biological replicate) of WT and ∆spoIIIL, ∆ssdC and ∆spoIIIL ∆ssdC mutant forespores during a sporulation time-course. (E) Representative images of ∆ssdC and ∆spoIIIL ∆ssdC mutant sporangia expressing CFP (under the control of σF-dependent promoter) and YFP (under the control of a control of σG-dependent promoter) at T3.5 and T5. Merged images show membrane in red and YFP in cyan. Scale bar is 2 μm. (F) Mean intensity (± STDEV of 3 biological replicates; n > 1000, for each biological replicate) of σG-dependent YFP signal in WT and ∆spoIIIL, ∆murAB and ∆spoIIIL ∆murAB mutant forespores, during a sporulation time-course. (G) Mean aspect ratio (± STDEV of 3 biological replicates; n > 500, for each biological replicate) of WT and ∆spoIIIL, ∆murAB and ∆spoIIIL ∆murAB mutant forespores, during a sporulation time-course. (H) Representative images of ∆murAB and ∆spoIIIL ∆murAB mutant sporangia expressing CFP (under the control of σF-dependent promoter) and YFP (under the control of a control of σG-dependent promoter) at T3.5 and T5. Merged images show membrane in red and YFP in cyan. Scale bar is 2 μm. (TIF) [file pgen.1011768.s007.tif]

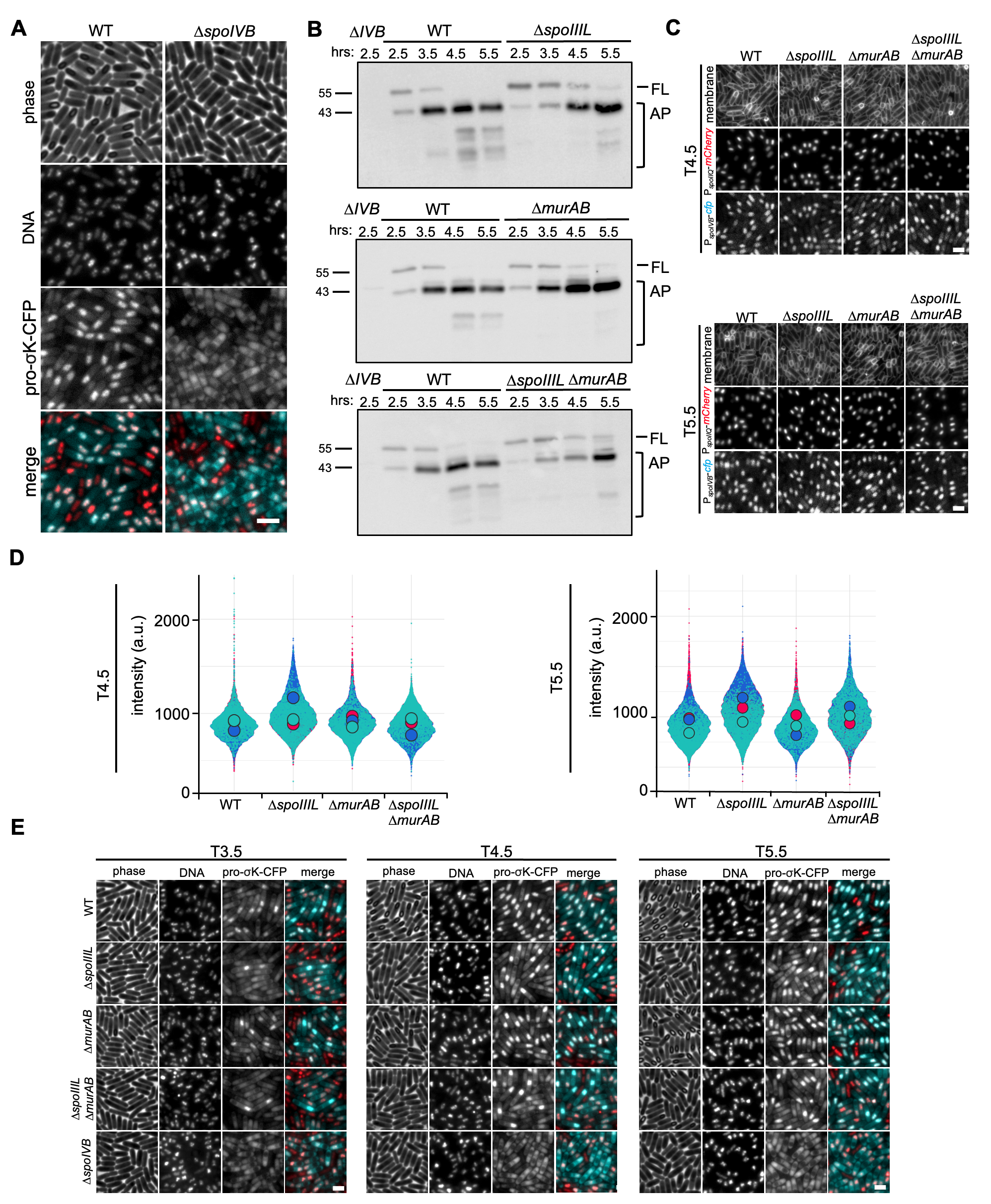

Supplement: S3 Fig — (A) Representative images of pro-σK-CFP localization at 4.5 (T4.5) hours after the onset of sporulation in WT and ∆spoIVB. Merge shows DNA stained with DAPI in red and pro-σK-CFP in cyan. Scale bar is 2 μm. (B) Uncropped Immunoblots comparing SpoIVB levels in WT and various mutants. Full-length (FL) and autoproteolyzed (AP) forms of SpoIVB are pointed out on the right and the position of molecular weight markers are shown to the left. (C) Representative images of PspoIVB-cfp reporter at 4.5 (T4.5) and 5.5 (T5.5) hours after the onset of sporulation in WT and ∆spoIIIL, ∆murAB, ∆spoIIIL ∆murAB mutant backgrounds. PspoIIQ-mCherry was used to identify forespores for quantitative image analysis of PspoIVB-cfp signal. Scale bar is 2 μm. (D) Mean intensity (± STDEV of 3 biological replicates; n > 500, for each biological replicate) of PspoIVB-cfp transcriptional reporter signal in WT and ∆spoIIIL, ∆murAB and ∆spoIIIL ∆murAB mutant forespores, at T4.5 (left) and T5.5 (right). (E) Representative images of pro-σK-CFP localization at 3.5 (T3.5), 4.5 (T4.5) and 5.5 (T5.5) hours after the onset of sporulation in WT and ∆spoIIIL, ∆murAB, ∆spoIIIL ∆murAB and ∆spoIVB mutant backgrounds. Merged shows DNA stained with DAPI in red and pro-σK-CFP in cyan. Scale bar is 2 μm. (TIF) [file pgen.1011768.s008.tif]

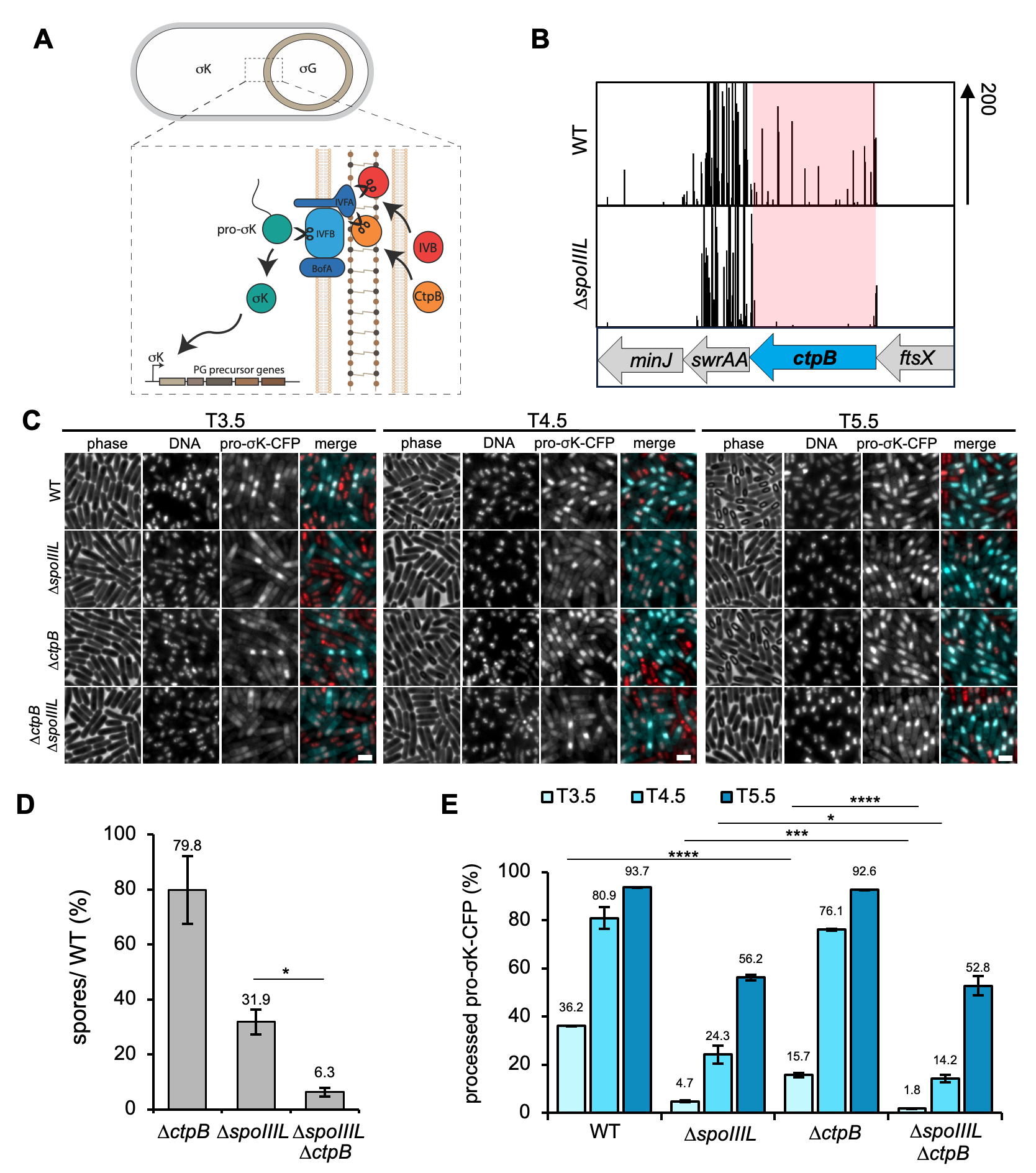

Supplement: S4 Fig — (B) Tn-seq profiles at the ctpB locus of WT and ∆spoIIIL mutant cells following 30 h of growth and sporulation in exhaustion medium. The height of the vertical lines represents the number of sequencing reads at each position (scale is 200 sequencing reads). Shaded regions highlight the significant reduction in sequencing reads at the ctpB locus. (C) Representative images of pro-σK-CFP localization at 3.5 (T3.5), 4.5 (T4.5) and 5.5 (T5.5) hours after the onset of sporulation in WT and ∆spoIIIL, ∆ctpB and ∆spoIIIL ∆ctpB mutant backgrounds. Scale bar is 2 μm. (D) Sporulation efficiency (% spores relative to WT) of ∆ctpB, ∆spoIIIL and ∆spoIIIL ∆ctpB mutants (n = 3, mean ± STDEV; *p < 0.05, Tukey’s multiple comparisons test). (E) Mean frequency of processed pro-σK-CFP at 3.5, 4.5 and 5.5 hours after the onset of sporulation in WT and ∆spoIIIL, ∆ctpB and ∆spoIIIL ∆ctpB mutant backgrounds (errors bars are ± STDEV of 3 biological replicates, > 100 cells for each biological replicate; *p < 0.05, ***p < 0.001, ****p < 0.0001, Tukey’s multiple comparisons test). (TIF) [file pgen.1011768.s009.tif]

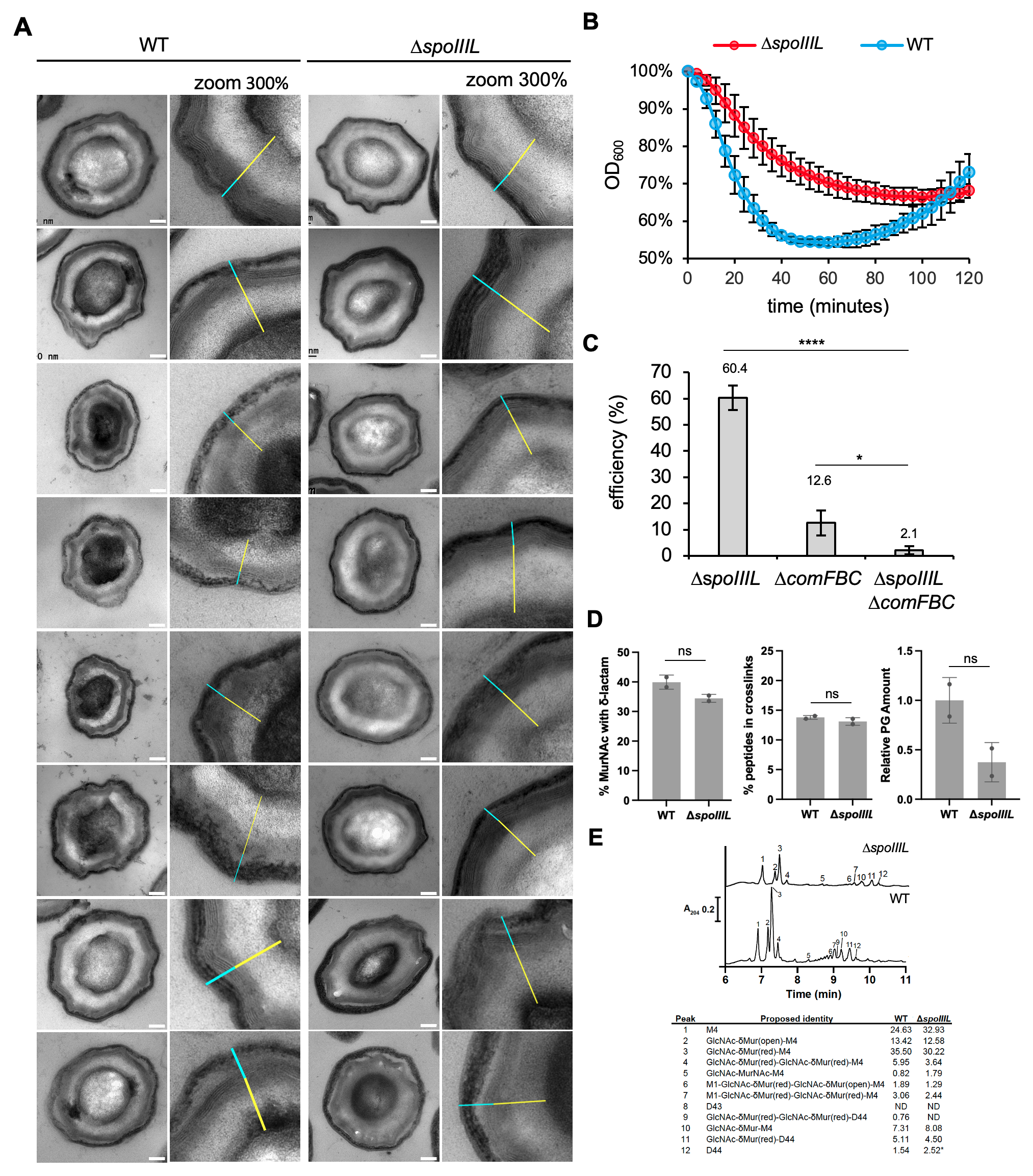

Supplement: S5 Fig — (A) Representative electron micrographs of mature spore sections of the WT and and ∆spoIIIL mutant. The yellow line highlights the region occupied by the cortex and the blue line the region occupied by the coat. Scale bar is 100 nm. (B) Germination efficiency of WT (blue) and ∆spoIIIL mutant (red) spores. Purified, mature spores of each strain were inoculated into LB broth at 37ºC, with constant agitation (200 rpm) to an OD600 of 1.2 (100%) and OD changes were monitored overtime (n = 3, ± STDEV). The ∆spoIIIL mutant exhibits a delay in germination, as demonstrated by the less pronounced reduction in OD overtime. (C) Mean transformation efficiency (% relative to WT) of the ∆spoIIIL, ∆comFBC and ∆spoIIIL ∆comFBC mutants (n = 3, ± STDEV; *p < 0.05, *p < 0.0001, Tukey’s multiple comparisons test). (D) Spore PG in WT and ∆spoIIIL mutant. Left panels shows % muramic acid moieties present as muramic δ-lactam; middle panels shows % peptide stems in crosslinks and right panel shows relative PG amount obtained from spore samples, determined by total area under identified peaks. (E) UPLC UV chromatogram of muramidase-digested PG obtained from WT and ∆spoIIIL mutant spores, with identified peaks numbered (top panel); (n = 2, mean ± STDEV; ns - not statistically significant, Tukey’s multiple comparisons test). Proposed identities and structures of identified peaks determined by UPLC-MS (bottom panel). (TIF) [file pgen.1011768.s010.tif]
